# Identification of Prognostic Biomarkers and Correlation With Immune Infiltrates in Hepatocellular Carcinoma Based on a Competing Endogenous RNA Network

**DOI:** 10.3389/fgene.2021.591623

**Published:** 2021-05-20

**Authors:** Zhangya Pu, Yuanyuan Zhu, Xiaofang Wang, Yun Zhong, Fang Peng, Yiya Zhang

**Affiliations:** ^1^Department of Infectious Diseases, Hunan Key Laboratory of Viral Hepatitis, Xiangya Hospital, Central South University, Changsha, China; ^2^NHC Key Laboratory of Cancer Proteomics, Xiangya Hospital, Central South University, Changsha, China; ^3^National Clinical Research Center for Geriatric Disorders, Xiangya Hospital, Central South University, Changsha, China; ^4^Department of Dermatology, Xiangya Hospital, Changsha, China

**Keywords:** hepatocellular carcinoma, competing endogenous RNA network, immune infiltration, prognostic prediction model, biomarkers

## Abstract

**Background:**

Hepatocellular carcinoma (HCC) is one of the most common malignant tumors worldwide. Recently, competing endogenous RNAs (ceRNA) have revealed a significant role in the progression of HCC. Herein, we aimed to construct a ceRNA network to identify potential biomarkers and illustrate its correlation with immune infiltration in HCC.

**Methods:**

RNA sequencing data and clinical traits of HCC patients were downloaded from TCGA. The limma R package was used to identify differentially expressed (DE) RNAs. The predicted prognostic model was established using univariate and multivariate Cox regression. A K-M curve, TISIDB and GEPIA website were utilized for survival analysis. Functional annotation was determined using Enrichr and Reactome. Protein-to-protein network analysis was implemented using SRTNG and Cytoscape. Hub gene expression was validated by quantitative polymerase chain reaction, Oncomine and the Hunan Protein Atlas database. Immune infiltration was analyzed by TIMMER, and Drugbank was exploited to identify bioactive compounds.

**Results:**

The predicted model that was established revealed significant efficacy with 3- and 5-years of the area under ROC at 0.804 and 0.744, respectively. Eleven DEmiRNAs were screened out by a K-M survival analysis. Then, we constructed a ceRNA network, including 56 DElncRNAs, 6 DEmiRNAs, and 28 DEmRNAs. The 28 DEmRNAs were enriched in cancer-related pathways, for example, the TNF signaling pathway. Moreover, six hub genes, CEP55, DEPDC1, KIF23, CLSPN, MYBL2, and RACGAP1, were all overexpressed in HCC tissues and independently correlated with survival rate. Furthermore, expression of hub genes was related to immune cell infiltration in HCC, including B cells, CD8^+^ T cells, CD4^+^ T cells, monocytes, macrophages, neutrophils, and dendritic cells.

**Conclusion:**

The findings from this study demonstrate that CEP55, DEPDC1, KIF23, CLSPN, MYBL2, and RACGAP1 are closely associated with prognosis and immune infiltration, representing potential therapeutic targets or prognostic biomarkers in HCC.

## Introduction

Hepatocellular carcinoma (HCC) is one of the most universally malignant tumors in the world, with increasing morbidity and mortality (Shi et al., 2016; Li et al., 2017). Currently, it is the fifth most common cancer and the fourth leading cause of cancer-related death worldwide (Long et al., 2019; Gu et al., 2020). Tumorigenesis of HCC is correlated with several liver primary diseases, such as viral infections, including Hepatitis B virus (HBV), Hepatitis C virus (HCV), and other kinds of hepatotropic viruses, as well as alcoholic liver diseases, dietary aflatoxin exposure diabetes, and other diseases (Fu et al., 2018; Li et al., 2019a). Despite continuous improvement in the methods of diagnosis and treatment, HCC remains a global clinical challenge due to its poor prognosis and low rate of 5-year survival (Yue et al., 2019; Zhang R. et al., 2020). Therefore, individual strategies based on identifying early potential prognostic biomarkers and novel therapeutic targets are urgently needed.

The correlation between protein-coding messenger RNA (mRNA) and non-coding RNA (ncRNA), including long non-coding RNAs (lncRNA) and microRNAs (miRNAs), is complicated and obscure. In 2011, the competing endogenous RNA (ceRNA) hypothesis was elucidated for the first time by Salmena et al. (2011) demonstrating that ncRNAs not only directly take part in the regulation of targeted gene expression but also absorb corresponding miRNAs as natural sponges due to their typically containing more than one miRNA response element (MRE) that competes with mRNA. Recently, increasing evidence indicates that the regulatory network comprised of lncRNA-miRNA-mRNA plays an important role in the physiology and development of various tumors, including HCC, gallbladder cancer, gastric cancer, and others (Long et al., 2019; Yue et al., 2019; Gu et al., 2020). Zhang et al. (2016) indicated that lncRNA-correlated ceRNA networks are involved in diverse biological cancer pathways in glioblastoma. Wang J.J. et al. (2019) identified six lncRNAs, including LINC00536 and MIR7-3HG, that have a significant effect on overall survival in breast cancer. Nevertheless, current studies based on ceRNA networks in multiple databases for HCC are insufficient.

Recently, increasing attention has been paid to immunotherapy research in various cancers, especially in advanced stages, including for mesothelioma, HCC, and others. However, the benefits from immunotherapies are diverse in various tumors and are difficult to evaluate due to a lack of trustworthy immune-related biomarkers (Pan et al., 2019; Chen et al., 2020). Immune-related cells infiltration into the tumor microenvironment (TME) is a key reason leading to immune responses at primary and secondary tumor sites, which is tightly regulated by various mediators, such as chemokines. Several studies have indicated that a variety of different immune cells, including CD4^+^ and CD8^+^T -cells, dendritic cells, and tumor-associated macrophages (TAMs), have been identified in different cancers, such as prostate cancer, HCC and others (Pan et al., 2019; Zhang Y. et al., 2020). Chen et al. (2015) also demonstrated that CD4^+^ and CD8^+^T-cells could be recruited into the TME after CXCR4 inhibition in sorafenib-treated HCC in a mouse model. Therefore, there is an impending requirement to identify potential predictors related to immune cell infiltration to enhance the efficacy of individual immunotherapeutic treatment in tumors.

**FIGURE 1:**
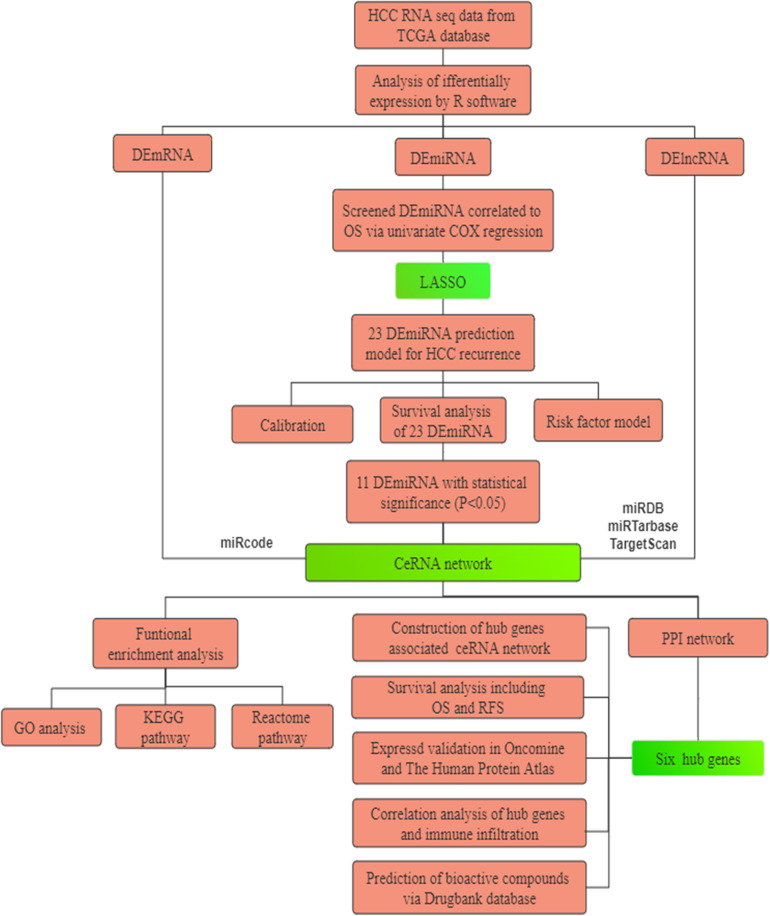
The flowchart of the present research. HCC: hepatocellular carcinoma. TCGA: The Cancer Genome Atlas. PPI: protein–protein interaction. GO: Gene Oncology. KEGG: Kyoto Encyclopedia of Genes and Genomes. ceRNA: competing endogenous RNAs. LASSO: the least absolute shrinkage and selection operator.

Study design is recapitulated in Figure 1. First, differentially expressed RNAs were analyzed in 371 cases of HCC and 50 normal liver tissues from The Cancer Genome Atlas (TCGA). Subsequently, a nomogram predicted model based on 23 miRNAs was established and revealed high performance. Next, we constructed a ceRNA network composed of 56 DELs, 6 DEMs, and 28 DEGs to illustrate preliminary interactions between mRNAs and ncRNAs. DEGs correlating to the ceRNA network were submitted for Gene Ontology (GO) and pathway enrichment analysis to clarify the underlying molecular mechanism in HCC. Finally, six hub genes, including CEP55, DEPDC1, CLSPN, KIF23, MYBL2, and RACGAP1, were identified by protein-to-protein (PPI) analysis and were all overexpressed in HCC samples with significant poor prognosis in HCC patients. Meanwhile they were also closely associated with immune infiltration in HCC. In summary, we believe these genes represent potential prognostic markers, immune-related biomarkers and immunological therapeutic targets for HCC treatment that deserved to be further explored in the future.

## Materials and Methods

### Data Acquisition From TCGA Database

A total of 371 HCC patients were included in this study. RNA sequencing (RNA-Seq), including lncRNA, mRNA (Illumina HiSeq RNA-Seq platform), and miRNA sequence data (Illumina HiSeq miRNA-Seq platform) from 371 HCC samples with survival data and 50 adjacent non-tumorous samples including the corresponding paired 50 HCC samples were downloaded from TCGA database (^1^ version 10.1, release time: May 15, 2019). Approval was not required by ethics committee, and the present study conformed to the publication guidelines by TCGA. The RNA sequence data were annotated based on the Ensemble gene ID. Log_2_ transformation was performed on all gene expression profiling. Then, Limma package (Version: 3.38.3) in R software (Version:3.5.2) was used to normalize the original data.

### Identification of Differentially Expressed (DE) RNAs

The expression profile of RNA sequencing data retrieved from TCGA was analyzed using the limma package of R software^2^ with the criterial of |log_2_ fold change| > 2 and the adjusted false discovery rate (FDR) of *P* < 0.05. Screened DE RNAs, including differentially expressed lncRNAs (DELs), differentially expressed miRNAs (DEMs), and differentially expressed mRNAs (DEGs), were used for subsequent analysis. Heat maps and volcano plots for DE RNAs were created using the heatmap package of R software.

### Univariate and Multivariate Cox Regression Analysis

Univariate Cox regression was used to screen for potential prognostic miRNAs correlated with overall survival in HCC patients. The least absolute shrinkage and selection operator (LASSO) detects the most influential variables because it analyzes all independent variables simultaneously. According to the principle of a penalty following a regularization path, the coefficients of less influential variables would trend toward zero. The glmnet package was used to perform LASSO algorithm with the criterial of *P* < 0.05. Multivariate Cox regression analysis via survival R package was utilized to establish a prognostic predictive model visualized by nomogram to show the correlation of the expression of DEMs and survival rate of specific HCC patients. The forest plot was created to display the results of multivariate Cox regression using the forestplot package.

### Evaluation of miRNA-Based Clinical Predictive Model

To evaluate the predictive performance of the prognostic model based on DEMs, first, a calibration curve of 3- and 5-year survival rates was determined to assess agreement between the predictive model and actual survival time. Moreover, the area under the curve (AUC) was calculated according to the time-dependent receiver operating characteristic analysis (ROC). Additionally, the risk score formula was performed to calculate total risk scores for individual patients based on the coefficient for each DEM. The risk score formula was built according to the following method: total risk score = sum of each coefficient × transcriptional expressed value of DEM. Then, HCC patients were divided into high- and low-risk groups by the median risk scores, regarded as the cutoff value. The difference in survival rate between the two groups was also evaluated. The correlation between expression levels of DEMs and OS in HCC patients was calculated by Kaplan-Meier (K-M) survival analysis using the survival package of R software according to the X tile method with a cutoff *P*-value < 0.05.

### Establishment of the ceRNA Regulatory Network

A co-expressed regulatory network comprised of DELs, DEMs, and DEGs was established to explore the potential functions of these DE RNAs in HCC. The interaction between DEMs and DELs was confirmed using the miRcode database^3^, which not only includes putative target sites of miRNAs from the integrated and searchable map but also contains conserved microRNA families annotated by the ENCyclopedia of DNA Elements (ENCODE) (Jeggari et al., 2012). DEM targets were predicted from three databases, including miRDB^4^, miRTarBase^5^, and TargetScan^6^ (Agarwal et al., 2015; Wong and Wang, 2015; Chou et al., 2018). Overlapping DEGs were selected for constructing the ceRNA network. Cytoscape^7^ software was used to visualize the expression correlation of DE RNAs.

### Functional Annotation and PPI Network Analysis

Gene Ontology (GO) analysis of differentially expressed genes, including biological process (BP), molecular function (MF) and cell components (CC), and Kyoto encyclopedia of genes and genomes (KEGG) pathway analysis, were enriched using the Enrich online tool^8^. The Reactome pathway was determined by the Reactome website^9^. The online STRING^10^ tool was used to construct the protein-to-protein (PPI) interaction network for DEGs involved in the ceRNA network and was visualized by Cytoscape. Hub genes were defined as the top six genes with the highest degree of connections to others via the CytoHubba plug-in of Cytoscape.

### Correlation of Hub Genes and Immune Infiltration Analysis

TIMMER is a comprehensive online database used for systematic analysis of the correlation of immune infiltration and gene markers of interest in 32 cancer types from TCGA^11^. The abundance of tumor-infiltrating immune cells (TIICs) from gene profiles is evaluated based on the statistical method of deconvolution published previously (Aran et al., 2015; Li et al., 2016). We analyzed expressed levels of hub genes in various tumors and the relationship between the expression of hub genes and immune infiltration, including B cells, CD4 + T cells, CD8 + T cells, neutrophils, macrophages, and dendritic cells. Furthermore, the correlation of hub gene expression and gene markers associated with tumor immune infiltration of monocytes, tumor associated macrophages (TAMs), M1 and M2 macrophages was performed via correlation modules. These gene markers were identified in previous studies (Sousa and Maatta, 2016; Danaher et al., 2017; Siemers et al., 2017). The strength of the correlation was evaluated by Spearman’s algorithm divided into five levels: very weak (0.00-0.19), weak (0.20-0.39), moderate (0.40-0.59), strong (0.60-0.79), and very strong (0.80-1.0). The cutoff criteria for statistical significance was *P*-value < 0.05. In addition, the SCNA module was used to identify differences in tumor infiltration of hub genes in HCC with different somatic copy number alterations.

## Results

### Identification of Differentially Expressed RNAs

**FIGURE 2:**
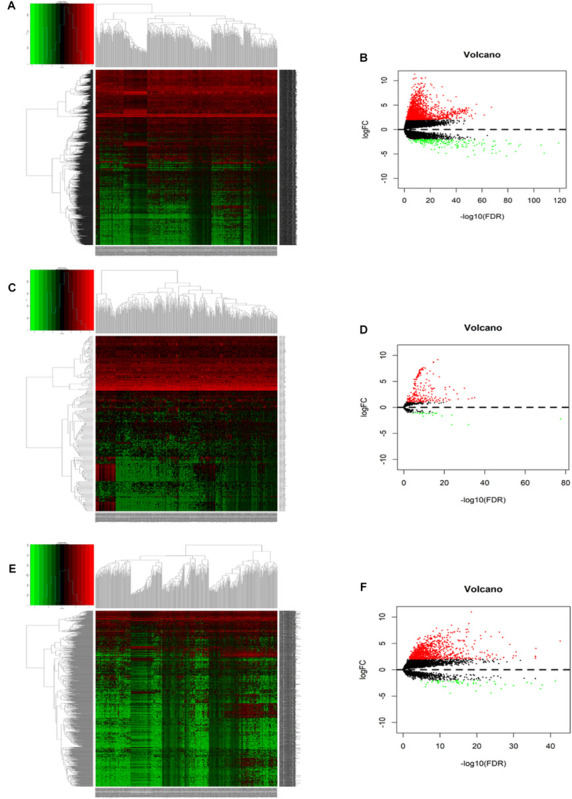
The hierarchical clustering heatmaps and volcano plots for all screened differentially expressed mRNA, miRNA and lncRNA in HCC based on TCGA data. Heatmaps located in the left panels represent differential expressed (DE) mRNAs **(A)**, miRNAs **(C)**, and lncRNAs **(E)**. Volcano plots located in the right panels indicate DEmRNAs **(B)**, DEmiRNAs **(D)**, and DElncRNAs **(F)** with the cutoff criteria of fold change ≥ 2 and *P*-value < 0.05. Red color: upregulated, green: downregulated, gray: not statistic expressed.

A total of 371 HCC samples and 50 non-tumor tissues were included in this study. Differentially expressed RNAs were analyzed using the limma R package with a screening cutoff threshold of |log_2_-fold change| > 2 and an adjusted *P*-value < 0.05. 1999 DEGs, 251 DEMs, and 1092 DELs were identified as differentially expressed RNAs. Among them, there were 1794 DEGs, 229 DEMs, and 1034 DELs upregulated. Moreover, differential expression of DEGs, DEMs and DELs is displayed by hierarchical clustering and volcano plots (Figure 2).

### Establishment of miRNA-Based Prognostic Predictive Model in HCC

**FIGURE 3:**
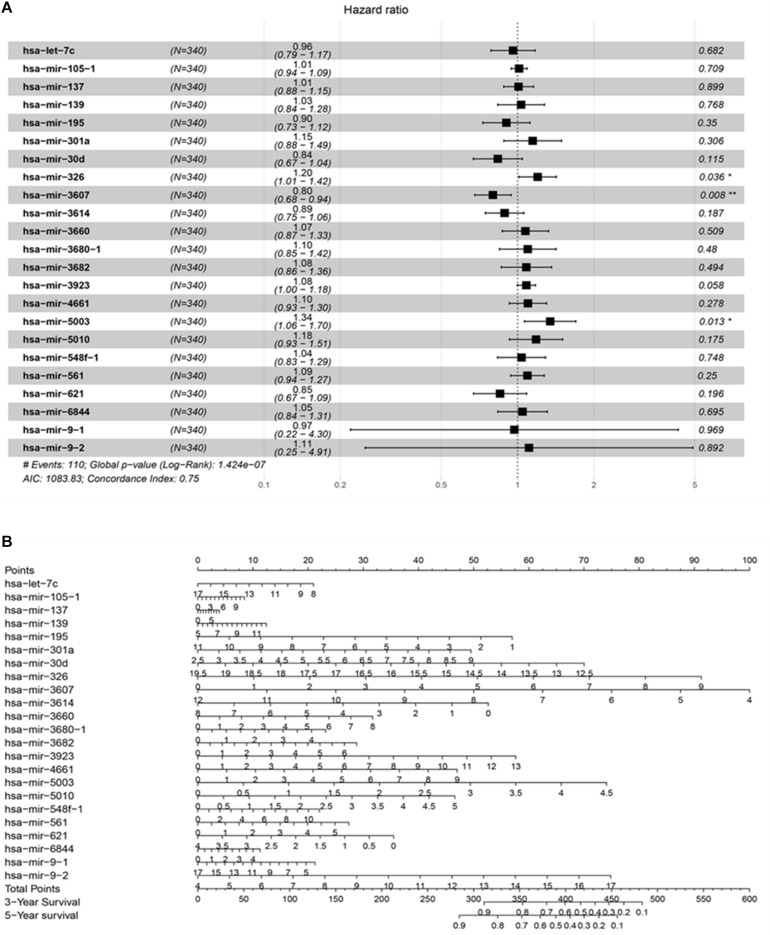
The prognostic predictive model for HCC. **(A)** The analyzed result of multivariate Cox proportional hazards regression involved with recurrence in TCGA HCC cohort. The middle point of the line indicates the hazard ratio (HR), and the whole length on behalf of the 95% CI for each DEmiRNA. **(B)** Nomogram based on differentially expressed miRNAs to predict survival in HCC asymptomatic individuals. The prognostic model aim to estimate the survival rate for individual patient, meanwhile reveal the upregulated or downregulated type for each miRNA. At first, draw a line straight upwards from each miRNA to obtain the points from the points axis. Repeat this step until the total scores were gained for 23 miRNAs. Then, after calculating the overall points according to the total points axis, draw a line straight down to the 3-year and 5-year survival axis from the location of total point axis based on the obtained overall scores to indicate the rate for the specific patient (for e.g., the 3-year survival rate is 60% if a patient get the total points of 400).

**FIGURE 4:**
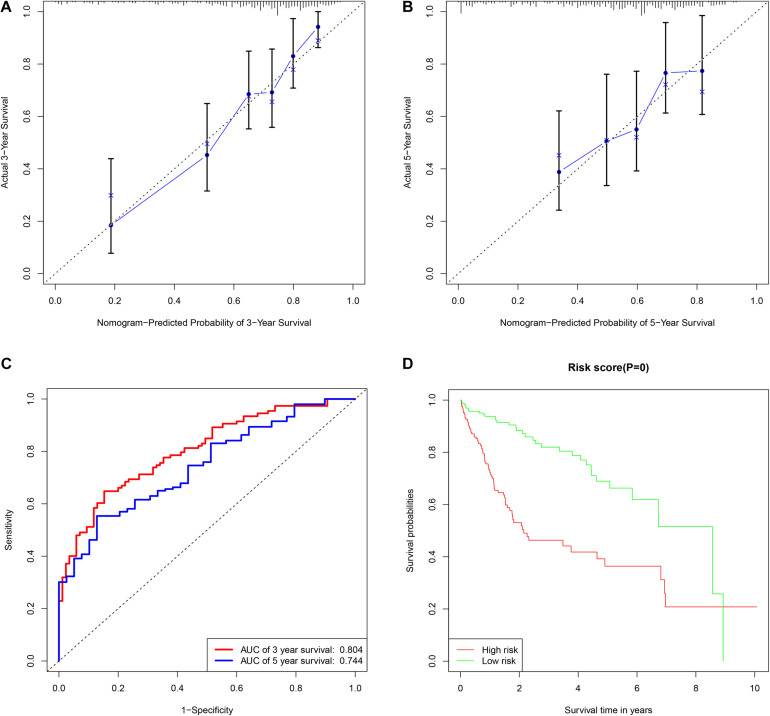
The assessment of miRNAs-based clinical prediction model. The calibration curves according to nomogram model to estimate the survival rate at 3-year **(A)** and 5-year **(B)**, the *X* and *Y*-axis represent predicted and actual survival time respectively. The efficacy of prognostic model of 3- and 5-years survival rate based on time dependent receiver operated characteristic curves **(C)**. The Kaplan-Meier curve of overall survival time between the high- and low- risk groups stratified by the mean of total risk scores **(D)**.

A total of 42 DEMs survival-related miRNAs were screened by univariate Cox regression from 251 DEMs identified in the HCC cohort (Supplementary Tables 1, 2). Next, these miRNAs were included in the LASSO analysis to calculate the corresponding coefficients. Twenty-three DEMs significantly correlated with survival were selected out (Supplementary Figure 1 and Supplementary Table 3). A simple-to-use nomogram predictive model was established to describe correlation of the expression of each miRNA and the 3- and 5-year overall survival rate of HCC patients based on multivariate Cox regression (Figure 3). Meanwhile, the 3- and 5-year calibration curves were drawn, showing good consistency between predicted survival probability and the actual survival rate (Figures 4A,B). The ROC curve also exhibited great reliability for the nomogram prediction model in discriminating tumors from normal tissues with the area under curve (AUC) of 3- and 5-year being 0.804 and 0.744, respectively (Figure 4C). Furthermore, the risk score (RS) of each patient in the HCC cohort was calculated, and the patients were subsequently divided into high-risk and low-risk groups according to the mean RS. K-M analysis indicated that patients in the high-risk group exhibited decreased survival compared to the low-risk group, with a log-rank *P*-value < 0.05 (Figure 4D).

### Construction of the lncRNA-miRNA-mRNA Regulatory Network

K-M analysis performed for 23 DEMs significantly related with OS revealed that 11 DEMs, including 8 that were upregulated, were independently statistically significant (Supplementary Table 4). Results showed that HCC patients with highly expressed DEMs had a shorter survival time than those with lower expression with a *P*-value < 0.05 (Supplementary Figure 2). Recently, increasing evidence has indicated that miRNAs play a significant role in the development and metastasis of tumors. To reveal potential signaling pathways regulated by these DEMs in HCC, the DIANA-miRPath database was exploited and revealed enrichment of cancer-related signaling pathways, such as PI3K-AKT, NF-kappa B, VEGF, and others (Supplementary Figure 3).

**FIGURE 5:**
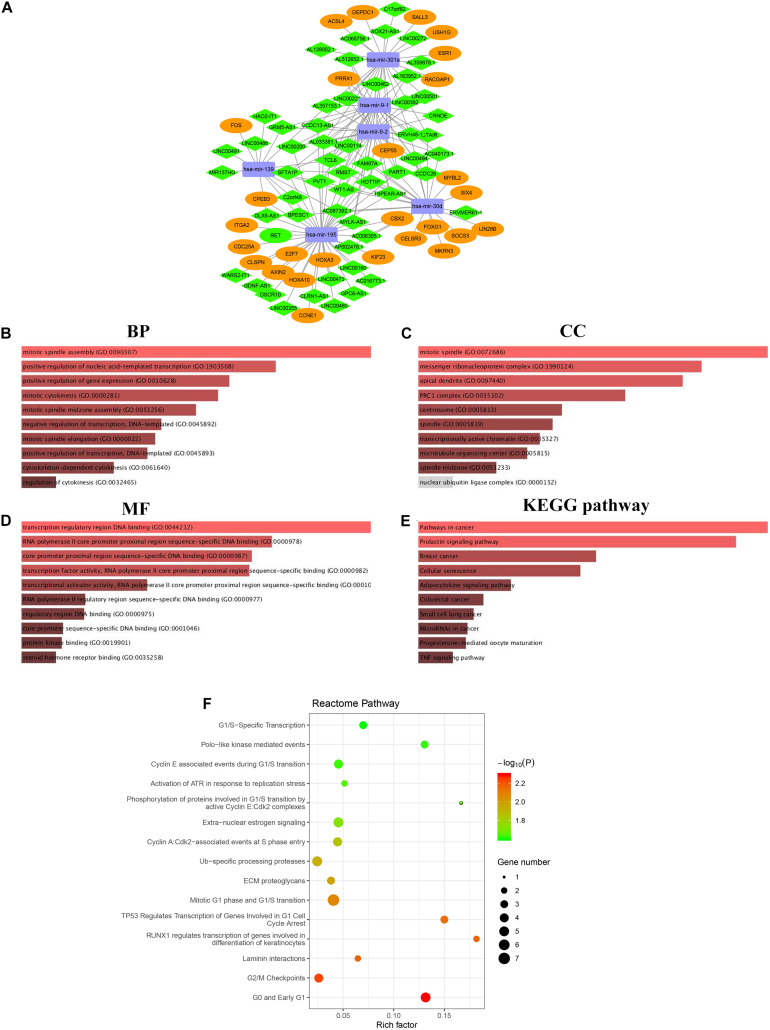
The lncRNA-miRNA-mRNA regulatory network in HCC cohort visualized by Cytoscape software 3.6.1 **(A).** Rectangle represent the 6 DEMs, diamond represent the 56 DELs, the ellipse represent the 28 DEGs. The functional enrichment analysis of DEGs correlated to ceRNA network **(B–F).** Top 10 biological process (BP) terms **(B)**. Top 10 cell components (CC) terms **(C)**. Top 10 molecular functions (MF) terms **(D)**. Top 10 significantly KEGG pathways **(E)**. Top 15 enriched Reactome pathways **(F)**.

The lncRNA targeted by the 11 DEMs was screened based on the interactions with 1092 DELs aforementioned. Six DEMs were targeted by 56 DELs according to the miRcode database. Next, targets of the 6 DEMs were predicted using miRTarBas, miRDB, and TargetScan databases. The overlapping 574 mRNAs predicted in all three databases were further intersected with 1999 DEGs identified in the HCC cohort, and only 28 DEGs existed in both groups (Supplementary Figure 4 and Supplementary Table 5). The representative interactions among 56 DELs, 6 DEMs, and 28 DEGs are summarized in Supplementary Table 6, and the ceRNA regulatory network, including gene nodes and preliminary interactions, was visualized using Cytoscape software (Figure 5A).

### Function and Pathway Enrichment Analysis of DEGs Involved in the ceRNA Network

Gene ontology and pathway enrichment analyses were performed to elucidate the functions of 28 DEGs in the ceRNA network correlated with the progression of HCC. Functional annotation of biological process (BP), cellular component (CC), and molecular function (MF), as well as KEGG pathway enrichment, were performed on the Enrichr comprehensive database, in which the top 10 highly enriched items for BP, CC, MF, and KEGG pathway are shown based on a *P*-value < 0.05 (Figures 5B-E). Notably, all top 10 items were closely related to cancer-related pathways, such as TNF signaling, breast cancer, small cell lung cancer and others. Moreover, Reactome pathway analysis was also developed to identify possible metabolic pathways in which the 28 DEGs are involved. A total of 28 pathways were identified, and the top 15 highly enriched pathways are presented in Figure 5F.

### Construction of PPI Network and Identification of Hub Genes

**FIGURE 6:**
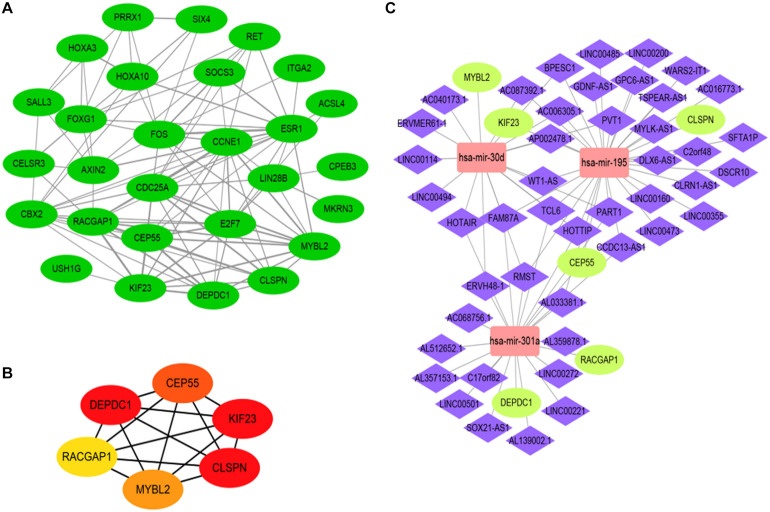
The construction of hub gene associated with ceRNA network based on analysis of protein to protein interaction (PPI) network. The PPI network of DEGs **(A)**. PPI network of 6 hub genes, the color of nodes from red to yellow indicates that the connected degrees between each molecule with others decrease gradually **(B)**. the hub gene ceRNA regulatory network including 3 DEMs (rectangle) and 6 hub DEGs (ellipse) as well as 46 lncRNA (diamond) **(C)**.

To further investigate the function of 28 DEGs associated with the ceRNA network at the protein level, we established a protein-to-protein interaction (PPI) network composed of 109 nodes and 218 degrees to visualize detailed interactions (Figure 6A). Considering the significance of hub genes in the ceRNA network, the CytoHubba plugin in Cytoscape software was exploited to identify hub genes by evaluating the number of degrees and connections. Finally, six hub genes, CEP55, DEPDC1, MYBL2, RACGAP1, CLSPN, and KIF23, were identified, which were all upregulated in HCC cohort (Figure 6B). The filled color of nodes from red to yellow indicates the degree of connectivity of hub genes with others gradually decreases. GO and pathway enrichment, including KEGG and Reactome analyses, were also performed (Supplementary Figure 5). Additionally, the sub ceRNA network, including 46 DELs, 3 DEMs (has-mir-30d, hasa-mir-195, has-mir-301a), and 6 hub genes, was built to delineate correlations among the DELs, DEMs, and hub genes (Figure 6C and Supplementary Table 7).

### Validation of Survival Analysis and Expression of Hub Genes

**FIGURE 7:**
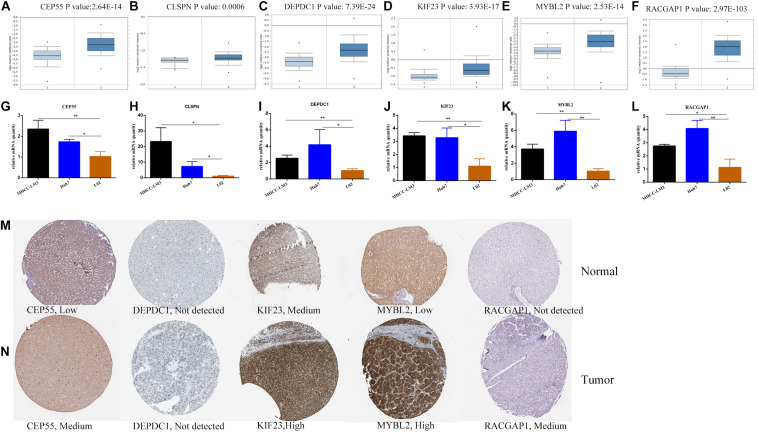
The transcriptional expression of six hub genes in HCC (right panels) and normal liver samples (left panels) based on Oncomine database **(A–F)**. The relative mRNA expression of six hub genes in HCC cell lines and liver cell line (G–L).*P < 0.05, **P < 0.01. The protein expression of hub genes based on Human Protein Atlas database **(M–N).** The protein expressed level of DEPDC1 was not detected in both tumor and normal liver tissues and the expressed data of CLSPN was lacking in database.

The correlation between expression of hub genes and OS (Supplementary Figures 6A-F) and recurrence-free survival (RFS) (Supplementary Figures 6G-L) was assessed using the GEPIA website. HCC patients with high expression levels of hub genes had a lower survival rate with respect to both OS and RFS. Among these, CEP55 had the highest prognostic *P*-value (0.00033 and 0.00063) correlated to overall and recurrence-free survival rates, respectively. Meanwhile, TISIDB tool that integrated five comprehensive public databases of HGNC, NCBI, Ensembl, Uniprot, and GeneCards was also exploited to evaluate the correlation to further confirm (Ru et al., 2019). It showed us consistent result that six hub genes were independently associated with poor prognosis in HCC patients with statistical significance (Supplementary Figures 6M–R). mRNA levels of hub genes in various tumor and normal tissues were examined using the TIMMER database. Results indicated that expression of hub genes was higher in various tumors than in corresponding normal tissues including breast cancer, colorectal cancer, gastric cancer and others (Supplementary Figure 7). Next, we validated transcriptional expression of hub genes in another HCC cohort from Oncomine database, which demonstrated overexpression of genes in the tumor group compared to non-tumor tissues as well with cutoff *P*-value < 0.01 (Figures 7A–F). Moreover, the mRNA expression of hub genes was detected in MHCC-LM3 and Huh7 HCC cell lines, and L02 normal liver cell line by quantitative polymerase chain reaction (qPCR), which showed the same conclusion with Oncomine database (Figures 7G–L). Additionally, immunohistochemical data from the Human Protein Atlas was used to verify protein expression of hub genes. Data for CLSPN was lacking in the database, and expression of DEPDC1 in both HCC and normal samples was not detected. However, the staining intensity or the range of positive areas of CEP55, KIF23, MYBL2, and RACGAP1 was higher in tumor samples (medium or high levels) than in non-tumor tissue (Figures 7M,N).

### Correlation of Hub Gene Expression and Immune Infiltration

**FIGURE 8:**
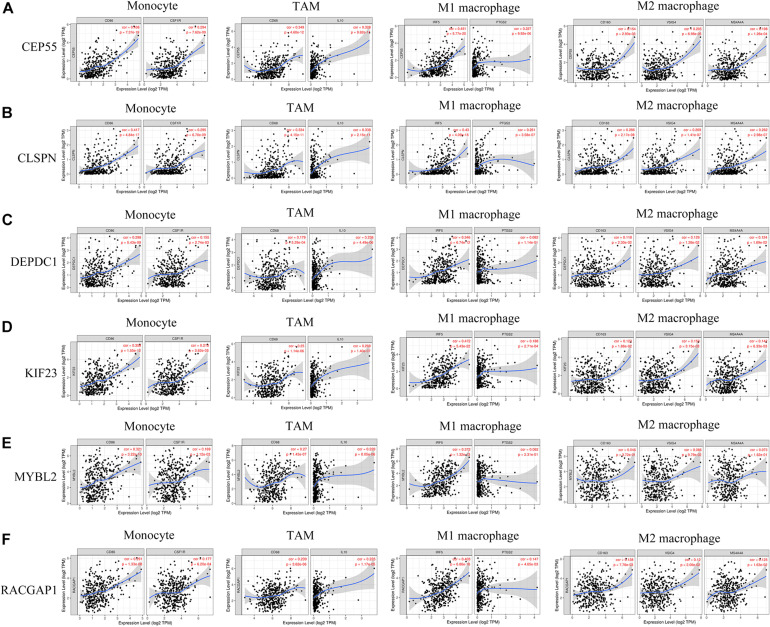
**(A–F)** Six hub genes expression correlated with macrophage polarization in HCC. Markers include CD86 and CSF1R of monocytes; CD68 and IL10 of TAMs (tumor-associated macrophages); IRF5 and PTGS2 of M1 macrophages; and CD163, VSIG4, and MS4A4A of M2 macrophages.

Recently, increasing evidence has demonstrated that tumor-infiltrating lymphocytes play a significant role in predicting lymph node status and survival in tumors. Therefore, we investigated whether expression of hub genes was related to immune-infiltrating levels in HCC using the TIMMER database. Results revealed that six hub genes were significantly related to immune infiltration of B cells, CD4^+^ T cells, CD8^+^T cells, macrophages, neutrophils, and dendritic cells in HCC, with statistical *P*-values of <0.0001. However, expression of CLSPN (cor = 0.08, *P* = 1.39e-01), CEP55 (cor = 0.018, *P* = 7.33e-01), and MYBL2 (cor = 0.098, *P* = 6.97e-02) had no significant correlation with tumor purity (Supplementary Figure 8). Next, to further investigate the correlation between expression of six hub genes and diverse immune infiltrating cells, the relationship between hub genes and a series of immune-related markers in various immune cells of HCC in TIMER database was focused. The analyzed immune markers included CD8^+^T cell, T cell (general), B cell, monocyte, tumor-associated macrophage (TAM), M1 Macrophage, M2 Macrophage, Neutrophils, Dendritic cell, Treg (Table 1). The results revealed that expressed levels of six hub genes were significantly related to most immune markers of various immune cell types. Importantly, we found that the expressed level of most markers of monocytes, TAM, M1and M2 macrophages showed strong correlation with hub genes of CEP55, CLSPN, DEPDC1, KIF23, and RACGAP1 except for MYBL2 which had no distinct relationship with gene markers of PTGS2, CD163, VSIG4, and MS4A4A (Table 1). Specially, we displayed that CD86, CSF1R of monocyte, CD68, IL10 of TAM, IRF5, PTSG2 of M1 macrophage, CD163, VSIG4, MS4A4A of M2 macrophage are significantly associated with hub genes in HCC (Figure 8). Furthermore, we also analyzed the correlation between hub genes and immune markers mentioned above of monocyte, TAM, M1 and M2 macrophage in HCC from GEPIA database. The correlation results are similar to those in TMER (Table 2). These findings suggest that hub genes are correlated with immune infiltration and may regulate macrophage polarization in HCC. Additionally, whether immune infiltrating levels of each immune subset are related to differential copy number of hub genes was also evaluated. However, no significant relationship was observed between most immune cells and hub genes (Supplementary Figure 9).

**TABLE 1:** The correlation between six hub genes and related genes and markers of immune cells for HCC in TIMER.

**Immune cell**	**Gene marker**	**CEP55**		**CLSPN**		**DEPDC1**		**KIF23**		**MYBL2**		**RACGAP1**	
		**Cor**	**P**	**Cor**	**P**	**Cor**	**P**	**Cor**	**P**	**Cor**	**P**	**Cor**	**P**
CD8^+^ T cell	CD8A	0.297	****	0.221	****	0.185	***	0.227	****	0.211	****	0.152	**
	CD8B	0.267	****	0.159	**	0.174	***	0.187	***	0.235	****	0.115	*
T cell	CD3D	0.372	****	0.209	****	0.24	***	0.273	****	0.365	****	0.152	**
(general)	CD3E	0.315	****	0.194	***	0.164	**	0.219	****	0.233	****	0.109	*
	CD2	0.323	****	0.197	***	0.171	***	0.23	****	0.254	****	0.104	*
B cell	CD19	0.32	****	0.237	****	0.238	****	0.263	****	0.309	****	0.221	****
	CD79A	0.279	****	0.14	**	0.108	*	0.16	**	0.201	****	0.061	0.238
Monocyte	CD86	0.438	****	0.417	****	0.298	****	0.358	****	321	****	0.291	****
	CSF1R	0.294	****	0.295	****	0.155	**	0.213	****	0.169	**	0.177	***
TAM	CD68	0.349	****	0.334	****	0.179	***	0.25	****	0.27	****	0.239	****
	IL10	0.328	****	0.338	****	0.236	****	0.269	****	0.229	****	0.225	****
M1 Macrophage	IRF5	0.451	****	0.43	****	0.346	****	0.472	****	0.372	****	0.403	****
	PTGS2	0.227	****	0.261	****	0.082	0.114	0.188	***	0.062	0.231	0.147	**
M2 Macrophage	CD163	0.154	**	0.286	****	0.118	*	0.122	*	0.046	0.372	0.138	**
	VSIG4	0.198	***	0.269	****	0.129	*	0.142	*	0.086	0.979	0.12	*
	MS4A4A	0.205	****	0.262	****	0.124	*	0.153	**	0.073	0.162	0.125	*
Neutrophils	ITGAM	0.403	****	0.457	****	0.324	****	0.369	****	0.284	****	0.312	****
	CCR7	0.192	***	0.163	**	0.05	0.341	0.11	*	0.078	0.133	0.056	0.285
Dendritic cell	HLA-DPB1	0.278	****	0.243	****	0.15	**	0.209	****	0.187	***	0.16	**
	HLA-DQB1	0.248	****	0.195	***	0.144	**	0.167	**	0.164	**	0.099	*
	HLA-DRA	0.209	****	0.303	****	0.188	***	0.23	****	0.166	**	0.201	****
	CD1C	0.205	****	0.171	***	0.063	0.23	0.156	**	0.116	*	0.115	*
	NRP1	0.319	****	0.417	****	0.185	***	0.324	****	0.118	*	0.442	**
	ITGAX	0.464	****	0.4444	****	0.322	****	0.404	*	0.35	****	0.314	****
Treg	FOXP3	0.201	***	0.338	****	0.236	****	0.222	****	0.094	0.0695	0.19	***
	CCR8	0.481	****	0.541	****	0.404	****	0.459	****	0.33	****	0.403	****
	STAT5B	0.203	****	0.49	****	0.266	*****	0.332	****	0.11	*	0.459	**
	TGFβ	0.418	****	0.307	****	0.195	***	0.334	****	0.308	****	0.271	****

Annotation: Cor, *R*-value of Spearman’s correlation; *P < 0.05; **P < 0.01; ***P < 0.001; and ****P < 0.0001.

**TABLE 2:** The correlation between six hub genes and gene markers of monocyte and macrophages in HCC from GEPIA.

**Immune cell type**		**Gene marker**	**CEP55**		**CLSPN**		**DEPDC1**		**KIF23**		**MYBL2**		**RACGAP1**	
			**R**	**P**	**R**	**P**	**R**	**P**	**R**	**P**	**R**	**P**	**R**	**P**
Tumor	Monocyte	CD86	0.53	*	0.44	*	0.3	****	0.52	*	0.25	****	0.38	****
		CSF1R	0.44	*	0.35	****	0.22	****	0.45	*	0.15	**	0.29	****
	TAM	CD68	0.33	****	0.3	****	0.18	***	0.33	****	0.2	***	0.27	****
		IL10	0.57	*	0.29	****	0.18	***	0.34	****	0.12	*	0.35	****
	M1 Macrophage	IRF5	0.34	****	0.35	****	0.27	****	0.34	****	0.36	****	0.38	****
		PTGS2	0.066	0.21	0.12	*	0.1	*	0.089	0.088	-0.013	0.8	0.048	0.36
	M2 Macrophage	CD163	0.46	*	0.26	****	0.16	**	0.43	*	0.13	*	0.24	****
		VSIG4	0.46	*	0.32	****	0.21	****	0.49	*	0.15	**	0.26	****
		MS4A4A	0.41	*	0.26	****	0.16	**	0.39	****	0.092	0.079	0.23	****
Normal	Monocyte	CD86	0.43	**	0.17	0.24	0.13	0.38	0.42	**	0.3	*	0.35	*
		CSF1R	0.41	**	0.26	0.064	0.1	0.48	0.53	****	0.33	*	0.42	**
	TAM	CD68	0.34	*	0.17	0.24	0.026	0.86	0.42	**	0.3	*	0.36	**
		IL10	0.25	0.07	0.1	0.47	0.02	0.89	0.19	0.2	0.15	0.3	0.36	*
	M1 Macrophage	IRF5	0.29	0.1	0.051	0.73	0.043	0.77	0.24	0.093	0.15	0.29	0.23	0.11
		PTGS2	0.02	0.89	-0.033	0.82	-0.098	0.5	0.064	0.66	0.00029	1	0.049	0.73
	M2 Macrophage	CD163	0.36	*	0.13	0.38	0.013	0.93	0.44	**	0.27	0.058	0.28	*
		VSIG4	0.2	0.15	0.097	0.5	0.00088	1	0.38	**	0.2	0.15	0.24	0.099
		MS4A4A	0.37	**	0.14	0.34	0.078	0.59	0.46	***	27	0.055	0.38	**

Annotation: Tumor, correlation analysis in HCC tissue of TCGA. Normal, correlation analysis in normal liver tissue of TCGA. R, *R*-value of Pearson’s correlation. *P < 0.05; **P < 0.01; ***P < 0.001; and ****P < 0.0001.

### Identification of Bioactive Compounds Targeting Hub Genes

Finally, we predicted potential bioactive compounds targeting hub genes using the Drugbank database, which is a comprehensive, freely accessible, online database including both drugs and drug target information. A total of 15 compounds targeting hub genes were identified, including CEP55 (Irdabisant, CEP-9722, CEP-1347, CEP-37440, Cefapirin), CLSPN (Calfactant, Calusterone), and MYBL2 (Clotrimazole, Propafenone, Letrozole, Sildenafil, Ranitidine, Valproic acid, Esomeprazole, and Pregabalin) (Table 3). Except for Calfactant, the 3D chemical structure of the other compounds is presented in Supplementary Figure 10. These results could provide new insight into potential novel therapeutic targets for HCC in the future.

**TABLE 3:** The predicted compounds target hub genes via Drugbank database.

**Hub genes**	**Predicted compounds**	**Accession number**	**Type**	**Classification in database**	**Chemical formula**	**Numbers of Clinical trial**
						
CEP55	Irdabisant	DB12900	small molecule	Investigational	C18H23N3O2	1
	CEP-9722	DB14882	small molecule	Investigational	C24H26N4O3	3
	CEP-1347	DB05403	small molecule	Investigational	C33H33N3O5S2	1
	CEP-37440	DB13060	small molecule	Investigational	C30H38ClN7O3	1
	Cefapirin	DB01139	small molecule	Approved	C17H17N3O6S2	Not available
CLSPN	Calfactant	DB06415	small molecule	Approved	not found	13
	Calusterone	DB01564	small molecule	Experimental	C21H32O2	Not available
MYBL2	Clotrimazole	DB00257	small molecule	Approved	C22H17ClN2	37
	Propafenone	DB01182	small molecule	Approved	C21H27NO3	22
	Letrozole	DB01006	small molecule	Approved, Investigational	C17H11N5	308
	Sildenafil	DB00203	small molecule	Approved, Investigational	C22H30N6O4S	290
	Ranitidine	DB00863	small molecule	Approved, Withdrawn	C13H22N4O3S	51
	Valproic acid	DB00313	small molecule	Approved, Investigational	C8H16O2	244
	Esomeprazole	DB00736	small molecule	Approved, Investigational	C17H19N3O3S	245
	Pregabalin	DB00230	small molecule	Approved, Investigational	C8H17NO2	306

*No compounds were detected in Drugbank database for hub genes of DEPDC1, KIF23, and RAGCAP1.*

## Discussion

Hepatocellular carcinoma is one of the most common malignant tumors in the world. In recent years, the prevalence of HCC is gradually increasing, especially in non-traditional high incidence areas, such as the United States and Europe. Most HCC patients are likely to be diagnosed at an advanced stage since HCC is asymptomatic at early stages, and effective biomarkers for early diagnosis and prognostic prediction are lacking (Lei et al., 2019; Li et al., 2019b; Liu et al., 2019). Currently, the main treatments for HCC include radiofrequency ablation, surgical resection, immunotherapy, and liver implantation, among others. However, the clinical efficacy of treatments for specific patients is not satisfactory due to poor therapeutic targets, tumor immune escape and complications, leading to a low 5-year survival rate (Yue et al., 2019; Zhang et al., 2019; Zhang Y. et al., 2020). In past decades, studies focused on immunotherapy for various cancers have obtained meaningful breakthroughs, especially in melanoma, non-small cell lung cancer, and others. Different types of immune cells infiltrate into the tumor microenvironment, which is a crucial reason for effective immune responses (Pan et al., 2019; Chen et al., 2020). It has been reported that immune cells, including natural killer cells, CD4^+^ and CD8^+^T-cells, TAMs, and dendritic cells, and others, are detected in cancer tissues, including HCC. Furthermore, these immune infiltrating cells are regulated by various mediators, such as chemokines. However, the mechanism of immune infiltration in the development of tumors is not completely understood (Sousa and Maatta, 2016; Siemers et al., 2017). Therefore, it is urgent and significant to elucidate the molecular mechanism and to identify immune-related signatures in HCC, which will aid in identifying new therapeutic targets and prognostic markers to increase the clinical efficacy and 5-year survival rate of HCC patients.

Currently, the ceRNA hypothesis of crosstalk between ncRNAs and mRNAs has received much attention and is considered a new measure of gene regulation at the posttranscriptional level, which provides new insight into revealing mechanisms of tumorigenesis and identifying potential diagnostic and prognostic biomarkers. A growing number of published studies have demonstrated that many predictive signatures are detected in various tumors based on ceRNA network analysis (Xiong et al., 2018; Wang M. et al., 2019; Xu et al., 2019; Wu et al., 2020; Xiao, 2020). MiRNAs, included in ncRNAs, are identified to be evolutionarily conserved, with an average length of 22-nt, and may bind to the 3′ untranslated region (3′UTR) of the targeted mRNAs according to the principle of complementary base pairing. An increasing body of evidence has demonstrated that dysregulated miRNAs play a crucial role in the initiation, progression, and therapy of various tumors (Long et al., 2019; Lou et al., 2019; Zhang et al., 2019). Baolei et al. revealed that miRNA-124 is a negative regulator of HCC with respect to proliferation and invasion by downregulating lncRNA-UCA1 (Zhao B. et al., 2019). Baltruskeviciene et al. (2017) found that downregulated expression of miRNA-148a and miRNA-625-3p is related to tumor budding in colorectal cancer, and EMT was considered a possible molecular mechanism.

In the present study, a prognostic predictive model was established based on 23 DEMs and exhibited great performance with the area under ROC, which was 0.804 for 3-year and 0.744 for 5-year survival. Moreover, we constructed a ceRNA network, including 56 DELs, 6 DEMs (hsa-mir-9-1,hsa-mir-9-2, hsa-mir-30d, hsa-mir-139, hsa-mir-195, hsa-mir-301a), and 28 DEGs, which identified several potential prognostic signatures for HCC. The finally screened 6 DEMs by ceRNA network were independently predicted factors related to poor prognosis in HCC patients. The major pathways that 6 DEMs participated were enriched in cancer-related regulatory signals. These findings here maybe give us a new avenue to early detect HCC patients, and evaluate clinical prognosis before or after receiving treatment via detecting the expressed level of specific DEMs mentioned above. U Lehmann et al. reported that hypermethylation of hsa-mir-9-1 is related to the development of breast cancer. Patients with pre-invasive intraductal lesions were detected by hypermethylated hsa-mir-9-1 (Lehmann et al., 2008). Notably, hsa-mir-195 belongs to the miR-195 family and is located on chromosome 17p13.1, and is correlated with proliferation and angiogenesis in prostate tumors by downregulating expression of the PRR11 gene (Cai et al., 2018). In Sannigrahi et al. (2017) research, they indicated that hsa-mir-139 was downregulated by HPV-16, leading to activation of HPV-16 oncogenic pathways and carcinogenesis of HPV-16 induced cervical and head and neck cancers. Hsa-mir-301a was found to play an oncogenic role in the occurrence and development of laryngeal squamous cell carcinoma (LSCC) by directly targeting the tumor suppressor gene Smad44 and downregulating its expression (Lu et al., 2015). Through analysis of SNP-array data generated from 8 medulloblastoma cell lines, Lu et al. (2009) found that hsa-mir-30d is overexpressed, however, the potentially involved biological processes and molecular mechanisms are still not clarified. Therefore, according to current studies, it has revealed that the 6 DEMs exhibited various biological functions in several cancer types, but the research progress is still limited especially in HCC. Further studies focused on both functions and molecular mechanisms in HCC were necessary. The discoveries in this study would be helpful to provide some clues for proceeding with the process.

In this study, six hub genes (including CEP55, DEPDC1, CLSPN, KIF23, MYBL2, and RACGAP1) were identified after comprehensive bioinformatic analysis in HCC cohort from TCGA, and were all overexpressed in HCC patients, which was validated in mRNA and protein expressed level based on various HCC cohorts from Oncomine and experimental data in HCC cell lines and normal liver cell line by qPCR method. Meanwhile, these hub genes were independently predicted factors associated with poor prognosis in HCC patients in various databases of GEPIA and TISIDB. The consistent results from databases and experimental approaches, to large extent, enhance the reliability of these findings. It suggested that the six hub genes would be potential biomarkers for evaluating clinical prognosis in HCC patients, and also provide some valuable clues for further study involved in the progression of HCC. Except for RACGAP1 that was the first time found to correlate to the progression in HCC, the other hub genes have also been reported in several tumors associated with biological process or regulatory pathways, although nowadays existing data is limited and more in-depth investigations are required. It was reported that CEP55 could promote proliferation and invasion in the progress of osteosarcoma. In another study, data showed that CEPP55 facilitates the EMT process in renal cell (RCC) cancer and participates in the AKT pathway (Xu et al., 2018; Chen et al., 2019). DEPDC1 has been identified in various cancers, including HCC, breast cancer, and prostate cancer, among others, and is positively involved with multiple tumorous biological processes, including proliferation, invasion etc. (Huang et al., 2017; Guo et al., 2019; Zhao H. et al., 2019). CLSPN was demonstrated to be overexpressed in RCC as assessed by immunohistochemistry in 95 RCC cases. It was also found that CLSPN activates the AKT signaling pathway and is co-expressed with several known tumor-related genes, such as programmed death ligand-1 (Kobayashi et al., 2020). Previous studies have reported that KIF23 is a kinesin-like motor protein and has two splice variants, KIF V1 and KIFV2, which are overexpressed in HCC samples but were not detected in non-tumor tissues. HCC patients with positive identified KIF V1 had a better overall 5-year survival rate than those with no KIF V1. However, there was no significant correlation between expression of KIF V2 and overall survival in patients (Sun et al., 2015; Wei et al., 2019). These discoveries by Xiaotong et al. conflicted with results in this study, indicating that additional investigations should be performed to further elucidate the role of KIF23 in the tumorigenesis of HCC (Sun et al., 2015). MYBL2 (MYB proto-oncogene like 2) is included in the family of MYB transcription factors and was overexpressed in breast cancer. Jianlin et al. revealed that overexpressed MYBL2 in breast cancer promotes growth and metastasis (Chen and Chen, 2018).

Additionally, in our study, we also found that all six hub genes were positively correlated with immune cell infiltration of B cells, CD4^+^ T cells, CD8^+^T cells, macrophages, neutrophils, and dendritic cells in HCC. Furthermore, our analysis revealed that the expressed level of hub genes was correlated with most immune-related markers of various immune cell types. Interestingly, hub genes of CEP55, CLSPN, DEPDC1, KIF23, and RACGAP1 were significantly related to markers of macrophages, showing its correlation to macrophage polarization. Until now, the correlation of six hub genes and immune infiltration in tumors has not been reported yet. Through an integrated bioinformatics analysis based on a ceRNA network, we identified several new biomarkers related to immune infiltration in HCC, which may represent potential prognostic and therapeutic targets. However, more in-depth experimental studies are required to further verify the function and elaborate on the underlying mechanisms in HCC. Finally, based on analysis of the Drugbank database, we found a total of 15 bioactive compounds targeting CEP55, CLSPN, and MYBL2, providing some new directions for drug development for HCC treatments in the future. Meanwhile, it suggested these genes could be potential values for predicting the efficacy of clinical treatment.

## Conclusion

In all, a simple-to-use nomogram predictive model was established based on miRNAs revealed great performance. We also constructed a ceRNA regulatory network to better understand the interactions between mRNAs and ncRNAs in HCC. Moreover, six hub genes were identified through PPI network analysis, all of which are overexpressed in HCC and are associated with survival. Besides, the expression of hub genes was independently correlated to poor prognosis in HCC patients, and was closely associated with immune infiltration in HCC. We believe these genes may be involved in the development of HCC and may represent potential prognostic biomarkers and individual therapeutic targets. However, further in-depth experiments are required to clarify detailed functions and mechanisms.

## Data Availability Statement

The original contributions presented in the study are included in the article/Supplementary Material, further inquiries can be directed to the corresponding author/s.

## Author Contributions

FP and YiZ designed the experiments and revised the manuscript. ZP analyzed the data and wrote the manuscript. XW, YunZ, and YuaZ searched and helped to analyze the data. All authors read and consent the final manuscript.

## Conflict of Interest

The authors declare that the research was conducted in the absence of any commercial or financial relationships that could be construed as a potential conflict of interest.

## Footnotes

1 https://portal.gdc.cancer.gov/

2 https://www.r-project.org/

3 http://www.mircode.org/

4 http://www.mirdb.org/

5 http://mirtarbase.mbc.nctu.edu.tw

6 http://www.targetscan.org

7 https://cytoscape.org/

8 http://www.enrichnet.org/

9 https://reactome.org/

10 https://string-db.org/

11 https://cistrome.shinyapps.io/timer/

## Abbreviations

lncRNAs: long non-coding RNAs
ceRNAs: competing endogenous RNAs
HCC: hepatocellular carcinoma
RFS: recurrence-free survival
OS: overall survival
TCGA: the Cancer Genome Atlas
DElncRNAs: differentially expressed lncRNA
DEmiRNAs: differentially expressed miRNA
DEmRNAs: differentially expressed mRNA
MREs: miRNA-response elements
GO: gene ontology
ROC: receiver operating characteristic
BP: biological process
CC: cellular component
MF: molecular function
EMT: Epithelial-mesenchymal transformation
RS: risk score.

## References

[B1] Agarwal V. Bell G. W. Nam J. W. Bartel D. P. (2015). Predicting effective microRNA target sites in mammalian mRNAs. *Elife* 4 299–316. 10.7554/eLife.05005 PMC4532895 26267216

[B2] Aran D. Sirota M. Butte A. J. (2015). Systematic pan-cancer analysis of tumour purity. *Nat. Commun.* 6:8971. 10.1038/ncomms9971 PMC4671203 26634437

[B3] Baltruskeviciene E. Schveigert D. Stankevicius V. Mickys U. Zvirblis T. Bublevic J. (2017). Down-regulation of miRNA-148a and miRNA-625-3p in colorectal cancer is associated with tumor budding. *BMC Cancer* 17:607. 10.1186/s12885-017-3575-z PMC5580437 28863773

[B4] Cai C. He H. Duan X. Wu W. Mai Z. Zhang T. (2018). miR-195 inhibits cell proliferation and angiogenesis in human prostate cancer by downregulating PRR11 expression. *Oncol. Rep.* 39 1658–1670. 10.3892/or.2018.6240 29393495 PMC5868402

[B5] Chen H. Zhu D. Zheng Z. Cai Y. Chen Z. Xie W. (2019). CEP55 promotes epithelial-mesenchymal transition in renal cell carcinoma through PI3K/AKT/mTOR pathway. *Clin. Transl. Oncol.* 21 939–949. 10.1007/s12094-018-02012-8 30607788

[B6] Chen J. Chen X. (2018). MYBL2 is targeted by miR-143-3p and regulates breast cancer cell proliferation and apoptosis. *Oncol. Res.* 26 913–922. 10.3727/096504017X15135941182107 29268817 PMC7844795

[B7] Chen J. Wang Z. Wang W. Ren S. Xue J. Zhong L. (2020). SYT16 is a prognostic biomarker and correlated with immune infiltrates in glioma: a study based on TCGA data. *Int. Immunopharmacol.* 84:106490. 10.1016/j.intimp.2020.106490 32289666

[B8] Chen Y. Ramjiawan R. R. Reiberger T. Ng M. R. Hato T. Huang Y. (2015). CXCR4 inhibition in tumor microenvironment facilitates anti-programmed death receptor-1 immunotherapy in sorafenib-treated hepatocellular carcinoma in mice. *Hepatology* 61 1591–1602. 10.1002/hep.27665 25529917 PMC4406806

[B9] Chou C. H. Shrestha S. Yang C. D. Chang N. W. Lin Y. L. Liao K. W. (2018). miRTarBase update 2018: a resource for experimentally validated microRNA-target interactions. *Nucleic Acids Res.* 46 D296–D302. 10.1093/nar/gkx1067 29126174 PMC5753222

[B10] Danaher P. Warren S. Dennis L. D’amico L. White A. Disis M. L. (2017). Gene expression markers of tumor infiltrating leukocytes. *J. Immunother. Cancer* 5:18. 10.1186/s40425-017-0215-8 PMC5319024 28239471

[B11] Fu S. Wang J. Hu X. Zhou R. R. Fu Y. Tang D. (2018). Crosstalk between hepatitis B virus X and high-mobility group box 1 facilitates autophagy in hepatocytes. *Mol. Oncol.* 12 322–338. 10.1002/1878-0261.12165 29316268 PMC5830655

[B12] Gu X. Li H. Sha L. Zhao W. (2020). Construction and comprehensive analyses of a competing endogenous RNA network in tumor-node-metastasis stage I hepatocellular carcinoma. *Biomed. Res. Int.* 2020:5831064. 10.1155/2020/5831064 PMC7036093 32104698

[B13] Guo W. Li H. Liu H. Ma X. Yang S. Wang Z. (2019). DEPDC1 drives hepatocellular carcinoma cell proliferation, invasion and angiogenesis by regulating the CCL20/CCR6 signaling pathway. *Oncol. Rep.* 42 1075–1089. 10.3892/or.2019.7221 31322256 PMC6667871

[B14] Huang L. Chen K. Cai Z. P. Chen F. C. Shen H. Y. Zhao W. H. (2017). DEPDC1 promotes cell proliferation and tumor growth via activation of E2F signaling in prostate cancer. *Biochem. Biophys. Res. Commun.* 490 707–712. 10.1016/j.bbrc.2017.06.105 28634077

[B15] Jeggari A. Marks D. S. Larsson E. (2012). miRcode: a map of putative microRNA target sites in the long non-coding transcriptome. *Bioinformatics* 28 2062–2063. 10.1093/bioinformatics/bts344 22718787 PMC3400968

[B16] Kobayashi G. Sentani K. Babasaki T. Sekino Y. Shigematsu Y. Hayashi T. (2020). Claspin overexpression is associated with high-grade histology and poor prognosis in renal cell carcinoma. *Cancer Sci.* 111 1020–1027. 10.1111/cas.14299 31912588 PMC7060467

[B17] Lehmann U. Hasemeier B. Christgen M. Muller M. Romermann D. Langer F. (2008). Epigenetic inactivation of microRNA gene hsa-mir-9-1 in human breast cancer. *J. Pathol.* 214 17–24. 10.1002/path.2251 17948228

[B18] Lei B. Zhou J. Xuan X. Tian Z. Zhang M. Gao W. (2019). Circular RNA expression profiles of peripheral blood mononuclear cells in hepatocellular carcinoma patients by sequence analysis. *Cancer Med.* 8 1423–1433. 10.1002/cam4.2010 30714679 PMC6488130

[B19] Li B. Severson E. Pignon J. C. Zhao H. Li T. Novak J. (2016). Comprehensive analyses of tumor immunity: implications for cancer immunotherapy. *Genome Biol.* 17:174. 10.1186/s13059-016-1028-7 PMC4993001 27549193

[B20] Li S. Huang Y. Huang Y. Fu Y. Tang D. Kang R. (2017). The long non-coding RNA TP73-AS1 modulates HCC cell proliferation through miR-200a-dependent HMGB1/RAGE regulation. *J. Exp. Clin. Cancer Res.* 36:51. 10.1186/s13046-017-0519-z PMC5389141 28403886

[B21] Li Y. Fu Y. Hu X. Sun L. Tang D. Li N. (2019a). The HBx-CTTN interaction promotes cell proliferation and migration of hepatocellular carcinoma via CREB1. *Cell Death Dis.* 10:405. 10.1038/s41419-019-1650-x PMC6538608 31138777

[B22] Li Y. Ma B. Yin Z. Liu P. Liu J. Li J. (2019b). Competing endogenous RNA network and prognostic nomograms for hepatocellular carcinoma patients who underwent R0 resection. *J. Cell. Physiol.* 234 20342–20353. 10.1002/jcp.28634 30963571

[B23] Liu J. Li W. Zhang J. Ma Z. Wu X. Tang L. (2019). Identification of key genes and long non-coding RNA associated ceRNA networks in hepatocellular carcinoma. *PeerJ.* 7:e8021. 10.7717/peerj.8021 PMC6827457 31695969

[B24] Long J. Bai Y. Yang X. Lin J. Yang X. Wang D. (2019). Construction and comprehensive analysis of a ceRNA network to reveal potential prognostic biomarkers for hepatocellular carcinoma. *Cancer Cell Int.* 19:90. 10.1186/s12935-019-0817-y PMC6458652 31007608

[B25] Lou W. Liu J. Ding B. Chen D. Xu L. Ding J. (2019). Identification of potential miRNA-mRNA regulatory network contributing to pathogenesis of HBV-related HCC. *J. Transl. Med.* 17:7. 10.1186/s12967-018-1761-7 PMC6317219 30602391

[B26] Lu Y. Gao W. Zhang C. Wen S. Huangfu H. Kang J. (2015). Hsa-miR-301a-3p acts as an oncogene in laryngeal squamous cell carcinoma via target regulation of Smad4. *J. Cancer* 6 1260–1275. 10.7150/jca.12659 26640587 PMC4643083

[B27] Lu Y. Ryan S. L. Elliott D. J. Bignell G. R. Futreal P. A. Ellison D. W. (2009). Amplification and overexpression of Hsa-miR-30b, Hsa-miR-30d and KHDRBS3 at 8q24.22-q24.23 in medulloblastoma. *PLoS One* 4:e6159. 10.1371/journal.pone.0006159 PMC2702821 19584924

[B28] Pan J. H. Zhou H. Cooper L. Huang J. L. Zhu S. B. Zhao X. X. (2019). LAYN Is a prognostic biomarker and correlated with immune infiltrates in gastric and colon cancers. *Front. Immunol.* 10:6. 10.3389/fimmu.2019.00006 PMC6362421 30761122

[B29] Ru B. Wong C. N. Tong Y. Zhong J. Y. Zhong S. S. W. Wu W. C. (2019). TISIDB: an integrated repository portal for tumor–immune system interactions. *Bioinformatics* 35 4200–4202. 10.1093/bioinformatics/btz210 30903160

[B30] Salmena L. Poliseno L. Tay Y. Kats L. Pandolfi P. P. (2011). A ceRNA hypothesis: the rosetta stone of a hidden RNA language? *Cell* 146 353–358. 10.1016/j.cell.2011.07.014 21802130 PMC3235919

[B31] Sannigrahi M. K. Sharma R. Singh V. Panda N. K. Rattan V. Khullar M. (2017). Role of host miRNA Hsa-miR-139-3p in HPV-16-induced carcinomas. *Clin. Cancer Res.* 23 3884–3895. 10.1158/1078-0432.CCR-16-2936 28143871

[B32] Shi L. Peng F. Tao Y. Fan X. Li N. (2016). Roles of long noncoding RNAs in hepatocellular carcinoma. *Virus Res.* 223 131–139. 10.1016/j.virusres.2016.06.008 27374059

[B33] Siemers N. O. Holloway J. L. Chang H. Chasalow S. D. Ross-Macdonald P. B. Voliva C. F. (2017). Genome-wide association analysis identifies genetic correlates of immune infiltrates in solid tumors. *PLoS One* 12:e0179726. 10.1371/journal.pone.0179726 PMC5531551 28749946

[B34] Sousa S. Maatta J. (2016). The role of tumour-associated macrophages in bone metastasis. *J. Bone Oncol.* 5 135–138. 10.1016/j.jbo.2016.03.004 27761375 PMC5063225

[B35] Sun X. Jin Z. Song X. Wang J. Li Y. Qian X. (2015). Evaluation of KIF23 variant 1 expression and relevance as a novel prognostic factor in patients with hepatocellular carcinoma. *BMC Cancer* 15:961. 10.1186/s12885-015-1987-1 PMC4682286 26674738

[B36] Wang J. J. Huang Y. Q. Song W. Li Y. F. Wang H. Wang W. J. (2019). Comprehensive analysis of the lncRNAassociated competing endogenous RNA network in breast cancer. *Oncol. Rep.* 42 2572–2582. 10.3892/or.2019.7374 31638237 PMC6826329

[B37] Wang M. Mao C. Ouyang L. Liu Y. Lai W. Liu N. (2019). Long noncoding RNA LINC00336 inhibits ferroptosis in lung cancer by functioning as a competing endogenous RNA. *Cell Death Differ.* 26 2329–2343. 10.1038/s41418-019-0304-y 30787392 PMC6889193

[B38] Wei B. Kong W. Mou X. Wang S. (2019). Comprehensive analysis of tumor immune infiltration associated with endogenous competitive RNA networks in lung adenocarcinoma. *Pathol. Res. Pract.* 215 159–170. 10.1016/j.prp.2018.10.032 30466766

[B39] Wong N. Wang X. (2015). miRDB: an online resource for microRNA target prediction and functional annotations. *Nucleic Acids Res.* 43 D146–D152. 10.1093/nar/gku1104 25378301 PMC4383922

[B40] Wu Z. H. Cai F. Zhong Y. (2020). Comprehensive analysis of the expression and prognosis for GBPs in head and neck squamous cell carcinoma. *Sci. Rep.* 10:6085. 10.1038/s41598-020-63246-7 PMC7142114 32269280

[B41] Xiao Y. (2020). Construction of a circRNA-miRNA-mRNA network to explore the pathogenesis and treatment of pancreatic ductal adenocarcinoma. *J. Cell Biochem.* 121 394–406. 10.1002/jcb.29194 31232492

[B42] Xiong D. D. Dang Y. W. Lin P. Wen D. Y. He R. Q. Luo D. Z. (2018). A circRNA-miRNA-mRNA network identification for exploring underlying pathogenesis and therapy strategy of hepatocellular carcinoma. *J. Transl. Med.* 16:220. 10.1186/s12967-018-1593-5 PMC6085698 30092792

[B43] Xu F. Zhao Y. Qin G. Huan Y. Li L. Gao W. (2019). Comprehensive analysis of competing endogenous RNA networks associated with cholangiocarcinoma. *Exp. Ther. Med.* 18 4103–4112. 10.3892/etm.2019.8052 31641385 PMC6796451

[B44] Xu L. Xia C. Sheng F. Sun Q. Xiong J. Wang S. (2018). CEP55 promotes the proliferation and invasion of tumour cells via the AKT signalling pathway in osteosarcoma. *Carcinogenesis* 39 623–631. 10.1093/carcin/bgy017 29579156

[B45] Yue C. Ren Y. Ge H. Liang C. Xu Y. Li G. (2019). Comprehensive analysis of potential prognostic genes for the construction of a competing endogenous RNA regulatory network in hepatocellular carcinoma. *Onco. Targets Ther.* 12 561–576. 10.2147/OTT.S188913 30679912 PMC6338110

[B46] Zhang K. Li Q. Kang X. Wang Y. Wang S. (2016). Identification and functional characterization of lncRNAs acting as ceRNA involved in the malignant progression of glioblastoma multiforme. *Oncol. Rep.* 36 2911–2925. 10.3892/or.2016.5070 27600337

[B47] Zhang R. Jiang Y. Y. Xiao K. Huang X. Q. Wang J. Chen S. Y. (2020). Candidate lncRNA-miRNA-mRNA network in predicting hepatocarcinogenesis with cirrhosis: an integrated bioinformatics analysis. *J. Cancer Res. Clin. Oncol.* 146 87–96. 10.1007/s00432-019-03090-z 31758243 PMC11804427

[B48] Zhang Y. Zhang L. Xu Y. Wu X. Zhou Y. Mo J. (2020). Immune-related long noncoding RNA signature for predicting survival and immune checkpoint blockade in hepatocellular carcinoma. *J. Cell. Physiol.* 235 9304–9316. 10.1002/jcp.29730 32330311

[B49] Zhang Z. Tang D. Wang B. Wang Z. Liu M. (2019). Analysis of miRNA-mRNA regulatory network revealed key genes induced by aflatoxin B1 exposure in primary human hepatocytes. *Mol. Genet. Genomic Med.* 7:e971. 10.1002/mgg3.971 PMC6825861 31502424

[B50] Zhao B. Lu Y. Cao X. Zhu W. Kong L. Ji H. (2019). MiRNA-124 inhibits the proliferation, migration and invasion of cancer cell in hepatocellular carcinoma by downregulating lncRNA-UCA1. *Onco Targets Ther.* 12 4509–4516. 10.2147/OTT.S205169 31354286 PMC6578570

[B51] Zhao H. Yu M. Sui L. Gong B. Zhou B. Chen J. (2019). High expression of DEPDC1 promotes malignant phenotypes of breast cancer cells and predicts poor prognosis in patients with breast cancer. *Front. Oncol.* 9:262. 10.3389/fonc.2019.00262 PMC6473048 31032225

