# Supplementary material for: Identification of Prognostic Biomarkers and Correlation With Immune Infiltrates in Hepatocellular Carcinoma Based on a Competing Endogenous RNA Network
Source: Front Genet. 2021 May 20;12:591623. doi: 10.3389/fgene.2021.591623 (PMC8173128; doi:10.3389/fgene.2021.591623)
Supplement: Supplementary file 11 [file Table_1.DOCX]

**Table S1**. Differentially expressed miRNA (DEMs) between HCC samples and nontumorous samples in TCGA cohort (total: 251 DEMs).

| **DEmiRNA** | **logFC** | **logCPM** | **PValue** | **FDR** | **Differential type** |
| --- | --- | --- | --- | --- | --- |
| hsa-mir-21 | 1.866094066 | 17.85925302 | 3.52E-38 | 1.16E-35 | UP |
| hsa-mir-93 | 1.736792336 | 12.57901424 | 9.37E-37 | 2.06E-34 | UP |
| hsa-mir-589 | 1.591054067 | 6.676439789 | 7.97E-35 | 1.19E-32 | UP |
| hsa-mir-10b | 3.660525892 | 14.25173239 | 9.91E-33 | 1.09E-30 | UP |
| hsa-mir-500a | 1.598379847 | 8.619071676 | 1.51E-29 | 1.43E-27 | UP |
| hsa-mir-4746 | 2.591834504 | 1.98300234 | 5.46E-29 | 4.50E-27 | UP |
| hsa-mir-224 | 3.328715919 | 7.53427591 | 2.41E-27 | 1.77E-25 | UP |
| hsa-mir-532 | 1.414837082 | 10.62562733 | 2.63E-25 | 1.45E-23 | UP |
| hsa-mir-183 | 3.957715909 | 11.86072483 | 4.63E-25 | 2.35E-23 | UP |
| hsa-mir-501 | 1.462410671 | 6.04919617 | 1.94E-24 | 9.14E-23 | UP |
| hsa-mir-103a-1 | 1.130874291 | 13.81971542 | 1.85E-23 | 7.81E-22 | UP |
| hsa-mir-103a-2 | 1.127777839 | 13.82184019 | 1.89E-23 | 7.81E-22 | UP |
| hsa-mir-452 | 2.53473522 | 8.120234349 | 2.67E-23 | 1.04E-21 | UP |
| hsa-mir-7706 | 1.756345124 | 1.55152106 | 5.78E-23 | 2.12E-21 | UP |
| hsa-mir-182 | 3.446814556 | 13.24716294 | 9.28E-23 | 3.22E-21 | UP |
| hsa-mir-96 | 3.839843871 | 3.781508522 | 1.04E-22 | 3.43E-21 | UP |
| hsa-mir-221 | 1.57358305 | 7.079533101 | 1.10E-21 | 3.29E-20 | UP |
| hsa-mir-660 | 1.324252575 | 6.466294933 | 1.35E-21 | 3.86E-20 | UP |
| hsa-mir-1180 | 1.761339504 | 4.610366269 | 5.37E-20 | 1.36E-18 | UP |
| hsa-mir-500b | 1.251445974 | 3.044686348 | 8.81E-20 | 2.15E-18 | UP |
| hsa-mir-1269a | 5.853026493 | 9.361284559 | 1.31E-19 | 3.08E-18 | UP |
| hsa-mir-34a | 1.449396129 | 8.585973405 | 3.81E-19 | 8.38E-18 | UP |
| hsa-mir-767 | 9.163857933 | 4.784214232 | 8.14E-19 | 1.73E-17 | UP |
| hsa-mir-502 | 1.060586312 | 3.94425429 | 1.52E-18 | 3.13E-17 | UP |
| hsa-mir-222 | 1.402010524 | 5.111224111 | 3.16E-18 | 6.14E-17 | UP |
| hsa-mir-1266 | 1.98719754 | 2.897264187 | 9.04E-18 | 1.66E-16 | UP |
| hsa-mir-454 | 1.158588373 | 3.136978557 | 1.25E-17 | 2.24E-16 | UP |
| hsa-mir-877 | 1.732075037 | 1.291196899 | 3.20E-17 | 5.41E-16 | UP |
| hsa-mir-34c | 3.698295228 | 2.269300291 | 5.14E-17 | 8.27E-16 | UP |
| hsa-mir-105-2 | 8.720228773 | 4.325907627 | 1.24E-16 | 1.95E-15 | UP |
| hsa-mir-105-1 | 8.635895124 | 4.32926991 | 1.69E-16 | 2.56E-15 | UP |
| hsa-mir-891a | 6.435924106 | 3.939345039 | 1.71E-16 | 2.56E-15 | UP |
| hsa-mir-9-1 | 3.600281942 | 8.666350826 | 2.51E-16 | 3.69E-15 | UP |
| hsa-mir-30d | 1.288524235 | 13.93333531 | 2.68E-16 | 3.85E-15 | UP |
| hsa-mir-9-3 | 3.589905258 | 8.67054409 | 2.85E-16 | 4.00E-15 | UP |
| hsa-mir-1301 | 1.363946195 | 3.53015321 | 3.89E-16 | 5.13E-15 | UP |
| hsa-mir-9-2 | 3.567063331 | 8.669445504 | 4.42E-16 | 5.72E-15 | UP |
| hsa-mir-3677 | 1.644785943 | 2.400142907 | 7.14E-16 | 8.73E-15 | UP |
| hsa-mir-196b | 3.473667535 | 6.467235173 | 1.11E-15 | 1.33E-14 | UP |
| hsa-mir-3200 | 2.531089954 | 2.163127551 | 1.21E-15 | 1.41E-14 | UP |
| hsa-mir-190b | 3.050684603 | 0.677792028 | 1.22E-15 | 1.41E-14 | UP |
| hsa-mir-4661 | 1.997228538 | 2.945685159 | 2.00E-15 | 2.27E-14 | UP |
| hsa-mir-3144 | 3.740704436 | 0.182666396 | 3.37E-15 | 3.77E-14 | UP |
| hsa-mir-3127 | 1.258143683 | 1.855180385 | 5.39E-15 | 5.93E-14 | UP |
| hsa-mir-301a | 1.315970189 | 3.289789811 | 6.45E-15 | 6.95E-14 | UP |
| hsa-mir-217 | 4.258760643 | 11.49516229 | 6.53E-15 | 6.95E-14 | UP |
| hsa-mir-4652 | 6.986218631 | 1.995705551 | 1.19E-14 | 1.21E-13 | UP |
| hsa-mir-19a | 1.554526855 | 5.99724186 | 1.25E-14 | 1.25E-13 | UP |
| hsa-mir-18a | 1.664020076 | 4.594753362 | 1.35E-14 | 1.33E-13 | UP |
| hsa-mir-1251 | 5.097907811 | 1.240952626 | 3.47E-14 | 3.28E-13 | UP |
| hsa-mir-3662 | 2.855743212 | 0.382521798 | 5.11E-14 | 4.69E-13 | UP |
| hsa-mir-135a-2 | 4.072911053 | 2.073225507 | 5.19E-14 | 4.69E-13 | UP |
| hsa-mir-4664 | 2.062697852 | 0.310964062 | 1.05E-13 | 9.04E-13 | UP |
| hsa-mir-20a | 1.310539285 | 9.568078349 | 1.32E-13 | 1.11E-12 | UP |
| hsa-mir-581 | 1.428680452 | 0.184181067 | 1.33E-13 | 1.11E-12 | UP |
| hsa-mir-1269b | 6.552833151 | 6.681970542 | 3.63E-13 | 2.92E-12 | UP |
| hsa-mir-552 | 5.369076301 | 6.155069385 | 7.21E-13 | 5.74E-12 | UP |
| hsa-mir-421 | 1.194463006 | 2.473194465 | 9.44E-13 | 7.42E-12 | UP |
| hsa-mir-643 | 1.352251696 | 0.475561088 | 9.66E-13 | 7.50E-12 | UP |
| hsa-mir-3591 | 2.261979051 | 1.849716846 | 1.18E-12 | 8.95E-12 | UP |
| hsa-mir-4326 | 1.633312062 | 2.624787156 | 1.22E-12 | 9.14E-12 | UP |
| hsa-mir-184 | 4.709706945 | 4.257721177 | 1.69E-12 | 1.26E-11 | UP |
| hsa-mir-301b | 1.816535823 | 1.060635444 | 2.43E-12 | 1.79E-11 | UP |
| hsa-mir-3923 | 7.523074791 | 3.947550032 | 2.81E-12 | 2.04E-11 | UP |
| hsa-mir-17 | 1.072463548 | 10.49743593 | 3.24E-12 | 2.33E-11 | UP |
| hsa-mir-135a-1 | 3.356115962 | 1.823224768 | 3.70E-12 | 2.62E-11 | UP |
| hsa-mir-760 | 1.775156676 | 0.242203885 | 5.11E-12 | 3.59E-11 | UP |
| hsa-mir-219b | 1.518496319 | -0.047977046 | 8.22E-12 | 5.71E-11 | UP |
| hsa-mir-216b | 4.116706487 | 5.925266502 | 1.17E-11 | 8.04E-11 | UP |
| hsa-mir-1270 | 2.257597389 | 1.409000818 | 1.44E-11 | 9.82E-11 | UP |
| hsa-mir-520a | 7.631655108 | 5.656513294 | 2.10E-11 | 1.40E-10 | UP |
| hsa-mir-2114 | 3.211710978 | 1.966342156 | 2.31E-11 | 1.52E-10 | UP |
| hsa-mir-939 | 1.243456797 | 0.445689566 | 2.35E-11 | 1.54E-10 | UP |
| hsa-mir-216a | 3.681213738 | 5.845646448 | 2.38E-11 | 1.54E-10 | UP |
| hsa-mir-362 | 1.062384608 | 4.929253855 | 2.65E-11 | 1.70E-10 | UP |
| hsa-mir-1254-1 | 2.298774599 | -0.617405757 | 2.86E-11 | 1.82E-10 | UP |
| hsa-mir-181b-2 | 1.018724281 | 6.851368841 | 3.78E-11 | 2.37E-10 | UP |
| hsa-mir-372 | 7.183513446 | 5.641194395 | 5.28E-11 | 3.23E-10 | UP |
| hsa-mir-4742 | 1.255760104 | 0.445165377 | 6.94E-11 | 4.17E-10 | UP |
| hsa-mir-34b | 3.296364437 | 0.465912125 | 7.45E-11 | 4.43E-10 | UP |
| hsa-mir-19b-2 | 1.062424825 | 6.962396619 | 7.77E-11 | 4.58E-10 | UP |
| hsa-mir-519a-1 | 7.352777249 | 5.108785571 | 9.04E-11 | 5.19E-10 | UP |
| hsa-mir-188 | 1.011941307 | 2.373656692 | 1.01E-10 | 5.73E-10 | UP |
| hsa-mir-765 | 1.850109028 | -0.277921893 | 1.07E-10 | 6.03E-10 | UP |
| hsa-mir-1226 | 1.706774829 | 0.862904415 | 1.09E-10 | 6.12E-10 | UP |
| hsa-mir-512-1 | 7.527835761 | 3.818384819 | 1.43E-10 | 7.78E-10 | UP |
| hsa-mir-520c | 7.245344014 | 2.386459997 | 2.08E-10 | 1.12E-09 | UP |
| hsa-mir-5010 | 1.133352197 | 0.560199532 | 2.47E-10 | 1.30E-09 | UP |
| hsa-mir-512-2 | 7.445623911 | 3.841972358 | 2.84E-10 | 1.48E-09 | UP |
| hsa-mir-937 | 1.667043347 | 1.732842063 | 3.73E-10 | 1.91E-09 | UP |
| hsa-mir-6783 | 2.042297118 | -0.603495159 | 5.47E-10 | 2.75E-09 | UP |
| hsa-mir-196a-2 | 4.673197949 | 3.259065595 | 6.28E-10 | 3.14E-09 | UP |
| hsa-mir-196a-1 | 4.600828395 | 3.120318411 | 7.31E-10 | 3.60E-09 | UP |
| hsa-mir-520b | 7.204872315 | 3.98975452 | 7.77E-10 | 3.80E-09 | UP |
| hsa-mir-5586 | 1.254264499 | 1.888039752 | 8.41E-10 | 4.08E-09 | UP |
| hsa-mir-548y | 3.935090945 | -0.303990029 | 1.02E-09 | 4.92E-09 | UP |
| hsa-mir-466 | 6.179620915 | 2.670274958 | 1.05E-09 | 5.03E-09 | UP |
| hsa-mir-520e | 6.646951345 | 1.890295006 | 1.18E-09 | 5.56E-09 | UP |
| hsa-mir-5003 | 1.611643075 | -0.29454556 | 1.18E-09 | 5.56E-09 | UP |
| hsa-mir-3682 | 1.154337561 | 1.022300534 | 1.25E-09 | 5.85E-09 | UP |
| hsa-mir-541 | 3.321174876 | 1.469489301 | 1.28E-09 | 5.93E-09 | UP |
| hsa-mir-6516 | 1.400788406 | 0.028326143 | 1.52E-09 | 6.90E-09 | UP |
| hsa-mir-526b | 6.487573702 | 5.654764456 | 1.55E-09 | 7.01E-09 | UP |
| hsa-mir-7974 | 2.392127979 | -0.405763658 | 1.66E-09 | 7.41E-09 | UP |
| hsa-mir-518f | 7.269383432 | 3.563597722 | 1.72E-09 | 7.60E-09 | UP |
| hsa-mir-518b | 6.590150387 | 4.05949306 | 2.06E-09 | 9.00E-09 | UP |
| hsa-mir-338 | 1.425623048 | 9.2610857 | 2.43E-09 | 1.03E-08 | UP |
| hsa-mir-519c | 6.750110592 | 2.425939298 | 2.93E-09 | 1.24E-08 | UP |
| hsa-mir-520f | 7.192230599 | 3.277170143 | 3.01E-09 | 1.27E-08 | UP |
| hsa-mir-519a-2 | 6.712897783 | 4.108374724 | 3.30E-09 | 1.37E-08 | UP |
| hsa-mir-516a-1 | 6.461374073 | 3.577881418 | 3.97E-09 | 1.64E-08 | UP |
| hsa-mir-1292 | 1.307716625 | -0.144215909 | 4.14E-09 | 1.70E-08 | UP |
| hsa-mir-516b-1 | 7.015054853 | 3.026859963 | 4.44E-09 | 1.79E-08 | UP |
| hsa-mir-200c | 2.780326327 | 8.486669747 | 4.83E-09 | 1.92E-08 | UP |
| hsa-mir-5187 | 1.238130655 | 0.27183635 | 5.32E-09 | 2.10E-08 | UP |
| hsa-mir-520h | 6.68015703 | 2.291356202 | 5.52E-09 | 2.17E-08 | UP |
| hsa-mir-520g | 6.91236341 | 3.123029557 | 5.96E-09 | 2.33E-08 | UP |
| hsa-mir-522 | 6.579322683 | 2.920660579 | 7.95E-09 | 3.09E-08 | UP |
| hsa-mir-130b | 1.046690303 | 5.731119021 | 9.95E-09 | 3.84E-08 | UP |
| hsa-mir-1323 | 6.089858683 | 2.553660475 | 1.11E-08 | 4.23E-08 | UP |
| hsa-mir-519d | 6.398551531 | 2.592963488 | 1.20E-08 | 4.54E-08 | UP |
| hsa-mir-517c | 6.190700677 | 2.256383623 | 1.40E-08 | 5.26E-08 | UP |
| hsa-mir-518e | 6.417362602 | 3.027274094 | 1.45E-08 | 5.44E-08 | UP |
| hsa-mir-1229 | 1.457845361 | 0.166467344 | 1.48E-08 | 5.50E-08 | UP |
| hsa-mir-431 | 2.209668292 | 5.006791416 | 1.55E-08 | 5.75E-08 | UP |
| hsa-mir-516a-2 | 5.916502288 | 3.330222464 | 1.56E-08 | 5.75E-08 | UP |
| hsa-mir-548x | 3.509505391 | -0.546841324 | 1.65E-08 | 6.04E-08 | UP |
| hsa-mir-1276 | 1.639770359 | -0.435640941 | 2.42E-08 | 8.69E-08 | UP |
| hsa-mir-653 | 1.684274184 | 3.882021512 | 2.65E-08 | 9.46E-08 | UP |
| hsa-mir-20b | 1.442833241 | 4.36196336 | 2.78E-08 | 9.87E-08 | UP |
| hsa-mir-520d | 6.045101819 | 2.000968945 | 2.98E-08 | 1.05E-07 | UP |
| hsa-mir-519b | 5.917655183 | 0.997237455 | 3.26E-08 | 1.15E-07 | UP |
| hsa-mir-549a | 2.265517723 | -0.654178385 | 3.48E-08 | 1.22E-07 | UP |
| hsa-mir-525 | 6.25224188 | 3.627069969 | 3.61E-08 | 1.26E-07 | UP |
| hsa-mir-3074 | 1.003884615 | 2.820543758 | 4.03E-08 | 1.39E-07 | UP |
| hsa-mir-137 | 4.06796214 | 0.554895043 | 4.11E-08 | 1.41E-07 | UP |
| hsa-mir-615 | 4.060171786 | 0.023794803 | 4.80E-08 | 1.64E-07 | UP |
| hsa-mir-498 | 6.050462453 | 1.596349265 | 5.96E-08 | 2.01E-07 | UP |
| hsa-mir-518a-1 | 6.02361634 | 2.063915204 | 6.00E-08 | 2.01E-07 | UP |
| hsa-mir-516b-2 | 5.837092368 | 1.984089359 | 7.00E-08 | 2.33E-07 | UP |
| hsa-mir-518c | 5.83419849 | 3.895713094 | 8.77E-08 | 2.89E-07 | UP |
| hsa-mir-521-1 | 5.677018021 | 1.116784605 | 9.25E-08 | 3.04E-07 | UP |
| hsa-mir-515-2 | 5.784339884 | 1.567560095 | 9.38E-08 | 3.06E-07 | UP |
| hsa-mir-6844 | 2.151366371 | -0.562662907 | 9.61E-08 | 3.11E-07 | UP |
| hsa-mir-524 | 5.339489439 | 0.952550638 | 1.32E-07 | 4.24E-07 | UP |
| hsa-mir-7705 | 1.144968332 | 0.259947854 | 1.39E-07 | 4.44E-07 | UP |
| hsa-mir-508 | 2.561959382 | 4.635372225 | 1.44E-07 | 4.60E-07 | UP |
| hsa-mir-527 | 5.807537635 | 2.02358074 | 1.60E-07 | 5.06E-07 | UP |
| hsa-mir-6716 | 1.353520631 | -0.37719971 | 1.61E-07 | 5.07E-07 | UP |
| hsa-mir-515-1 | 5.66052809 | 1.565308136 | 1.69E-07 | 5.29E-07 | UP |
| hsa-mir-1295a | 2.23680438 | 1.544362607 | 2.11E-07 | 6.54E-07 | UP |
| hsa-mir-3189 | 2.472299172 | -0.35086465 | 2.13E-07 | 6.57E-07 | UP |
| hsa-mir-1293 | 3.410173667 | -0.653021511 | 2.33E-07 | 7.15E-07 | UP |
| hsa-mir-514a-1 | 2.895587026 | 2.358629155 | 3.03E-07 | 9.26E-07 | UP |
| hsa-mir-509-3 | 3.047055842 | 1.904975233 | 3.29E-07 | 1.00E-06 | UP |
| hsa-mir-523 | 5.384028648 | 1.658508527 | 3.67E-07 | 1.11E-06 | UP |
| hsa-mir-1254-2 | 1.661912137 | -0.658599667 | 4.10E-07 | 1.24E-06 | UP |
| hsa-mir-548f-1 | 4.606963969 | 0.248887853 | 4.15E-07 | 1.24E-06 | UP |
| hsa-mir-519e | 4.213863903 | -0.236446965 | 5.37E-07 | 1.58E-06 | UP |
| hsa-mir-6715b | 2.269462948 | 2.367289604 | 5.65E-07 | 1.65E-06 | UP |
| hsa-mir-509-2 | 2.968564094 | 1.553843648 | 6.11E-07 | 1.78E-06 | UP |
| hsa-mir-92b | 1.126974974 | 4.243738916 | 6.36E-07 | 1.83E-06 | UP |
| hsa-mir-577 | 2.461011817 | 0.631194245 | 9.29E-07 | 2.66E-06 | UP |
| hsa-mir-205 | 4.230841471 | 3.377326793 | 9.32E-07 | 2.66E-06 | UP |
| hsa-mir-518a-2 | 5.391153854 | 2.134360351 | 9.69E-07 | 2.76E-06 | UP |
| hsa-mir-373 | 4.943055667 | 2.537623432 | 1.05E-06 | 2.96E-06 | UP |
| hsa-mir-521-2 | 3.628351306 | -0.502716383 | 1.11E-06 | 3.12E-06 | UP |
| hsa-mir-141 | 2.316355658 | 5.812034703 | 1.25E-06 | 3.49E-06 | UP |
| hsa-mir-3117 | 1.851125106 | -0.162166706 | 1.39E-06 | 3.86E-06 | UP |
| hsa-mir-1283-1 | 4.423933528 | 0.062369125 | 1.51E-06 | 4.18E-06 | UP |
| hsa-mir-514a-3 | 2.639312538 | 2.355539613 | 1.96E-06 | 5.41E-06 | UP |
| hsa-mir-3691 | 1.215717032 | -0.256817858 | 2.00E-06 | 5.51E-06 | UP |
| hsa-mir-6720 | 2.418664311 | -0.649820985 | 2.06E-06 | 5.59E-06 | UP |
| hsa-mir-509-1 | 2.808596202 | 1.539411661 | 2.23E-06 | 6.03E-06 | UP |
| hsa-mir-1283-2 | 4.524699491 | 0.478539107 | 2.37E-06 | 6.38E-06 | UP |
| hsa-mir-517a | 4.618606496 | 3.757269555 | 2.38E-06 | 6.38E-06 | UP |
| hsa-mir-517b | 4.6080496 | 3.747057444 | 2.44E-06 | 6.49E-06 | UP |
| hsa-mir-6734 | 1.219095217 | -0.638315234 | 2.98E-06 | 7.83E-06 | UP |
| hsa-mir-410 | 1.559158536 | 5.145162675 | 3.10E-06 | 8.12E-06 | UP |
| hsa-mir-371a | 5.369136303 | 1.874014797 | 3.31E-06 | 8.65E-06 | UP |
| hsa-mir-6854 | 1.015706957 | 0.011070332 | 3.40E-06 | 8.85E-06 | UP |
| hsa-mir-147b | 1.677965982 | -0.138180003 | 3.61E-06 | 9.35E-06 | UP |
| hsa-mir-412 | 1.775196423 | 4.077744543 | 3.72E-06 | 9.58E-06 | UP |
| hsa-mir-3680-1 | 1.408298587 | -0.644348237 | 3.84E-06 | 9.85E-06 | UP |
| hsa-mir-203b | 3.575226056 | 2.110671669 | 3.96E-06 | 1.01E-05 | UP |
| hsa-mir-526a-2 | 3.649106637 | -0.505655131 | 4.63E-06 | 1.18E-05 | UP |
| hsa-mir-1224 | 2.265404736 | 0.2959568 | 5.38E-06 | 1.36E-05 | UP |
| hsa-mir-6514 | 1.028283765 | -0.409301824 | 5.51E-06 | 1.39E-05 | UP |
| hsa-mir-432 | 1.669498453 | 4.653699584 | 5.84E-06 | 1.46E-05 | UP |
| hsa-mir-892a | 3.722319104 | -0.441529736 | 6.07E-06 | 1.52E-05 | UP |
| hsa-mir-944 | 1.531274054 | 1.466196424 | 6.34E-06 | 1.58E-05 | UP |
| hsa-mir-514a-2 | 2.524450645 | 2.353869768 | 6.94E-06 | 1.71E-05 | UP |
| hsa-mir-485 | 1.6503289 | 3.84679539 | 8.16E-06 | 1.99E-05 | UP |
| hsa-mir-561 | 2.565961815 | -0.266809771 | 1.20E-05 | 2.89E-05 | UP |
| hsa-mir-499a | 2.060765674 | 2.154011822 | 1.78E-05 | 4.22E-05 | UP |
| hsa-mir-3922 | 1.04576025 | -0.012000844 | 2.08E-05 | 4.86E-05 | UP |
| hsa-mir-493 | 1.416170407 | 5.00824419 | 2.20E-05 | 5.12E-05 | UP |
| hsa-mir-539 | 1.457088446 | 4.088528454 | 2.88E-05 | 6.65E-05 | UP |
| hsa-mir-3131 | 2.085615279 | -0.192118178 | 4.03E-05 | 9.18E-05 | UP |
| hsa-mir-3187 | 1.24992864 | -0.631754901 | 4.90E-05 | 0.000110736 | UP |
| hsa-mir-889 | 1.338176647 | 5.783424819 | 6.91E-05 | 0.000152565 | UP |
| hsa-mir-409 | 1.127168669 | 5.587680065 | 8.86E-05 | 0.000192773 | UP |
| hsa-mir-204 | 1.222631928 | 5.888672047 | 8.88E-05 | 0.000192773 | UP |
| hsa-mir-5683 | 1.189256525 | 1.14433015 | 0.000128655 | 0.00027391 | UP |
| hsa-mir-3660 | 2.862077303 | -0.731497014 | 0.00013017 | 0.000276246 | UP |
| hsa-mir-551b | 1.7790801 | 3.126779544 | 0.00015021 | 0.000317752 | UP |
| hsa-mir-496 | 1.32002079 | 2.679490629 | 0.00016943 | 0.000357264 | UP |
| hsa-mir-135b | 1.718625376 | 1.915300025 | 0.00030456 | 0.000616593 | UP |
| hsa-mir-380 | 1.416748934 | 1.651513573 | 0.000432125 | 0.000864249 | UP |
| hsa-mir-206 | 1.617942805 | 0.185750239 | 0.000475975 | 0.000949074 | UP |
| hsa-mir-483 | 1.869686778 | 8.460116606 | 0.000500573 | 0.000995116 | UP |
| hsa-mir-4739 | 1.228348811 | -0.107222357 | 0.000557555 | 0.001105063 | UP |
| hsa-mir-129-1 | 1.409668575 | 0.466795201 | 0.000580968 | 0.001144594 | UP |
| hsa-mir-129-2 | 1.343759242 | 0.538998091 | 0.000677708 | 0.001311694 | UP |
| hsa-mir-655 | 1.112470583 | 2.269885992 | 0.000931955 | 0.001766215 | UP |
| hsa-mir-506 | 2.185402888 | -0.325121117 | 0.00095362 | 0.001793131 | UP |
| hsa-mir-346 | 1.542780909 | -0.340028856 | 0.001148136 | 0.002128567 | UP |
| hsa-mir-31 | 1.657723736 | 1.069525236 | 0.001438078 | 0.00264382 | UP |
| hsa-mir-3174 | 1.034311815 | -0.713246549 | 0.001642458 | 0.002969924 | UP |
| hsa-mir-668 | 1.595187226 | -0.617682723 | 0.001731811 | 0.003105966 | UP |
| hsa-mir-323b | 1.121207055 | 2.921562012 | 0.001825219 | 0.003264619 | UP |
| hsa-mir-3681 | 1.743803911 | -0.060560555 | 0.002224592 | 0.00392575 | UP |
| hsa-mir-323a | 1.06787659 | 3.337811325 | 0.004928405 | 0.008340378 | UP |
| hsa-mir-599 | 1.551952473 | -0.454620897 | 0.005027733 | 0.008465061 | UP |
| hsa-mir-202 | 1.440696764 | -0.081025549 | 0.00515351 | 0.00865475 | UP |
| hsa-mir-1197 | 1.541840766 | -0.41944987 | 0.006776727 | 0.011153715 | UP |
| hsa-mir-1911 | 2.151389063 | 0.939190627 | 0.00721713 | 0.011761249 | UP |
| hsa-mir-211 | 1.21001746 | 0.689201976 | 0.01001811 | 0.01604669 | UP |
| hsa-mir-802 | 1.667287373 | 1.496234602 | 0.011348934 | 0.018005521 | UP |
| hsa-mir-424 | -2.222883362 | 7.416954299 | 5.62E-81 | 3.71E-78 | down |
| hsa-mir-1258 | -3.349226771 | 1.6201256 | 9.01E-35 | 1.19E-32 | down |
| hsa-mir-139 | -1.500161031 | 7.46632081 | 2.91E-26 | 1.92E-24 | down |
| hsa-mir-490 | -3.350719139 | 1.009669493 | 3.54E-26 | 2.12E-24 | down |
| hsa-mir-3607 | -1.656508447 | 6.435093514 | 4.97E-21 | 1.37E-19 | down |
| hsa-mir-450a-2 | -1.146616426 | 2.451406213 | 2.77E-18 | 5.53E-17 | down |
| hsa-mir-450a-1 | -1.120702113 | 2.438296228 | 4.91E-18 | 9.26E-17 | down |
| hsa-mir-511 | -1.151374548 | 3.971223009 | 2.04E-17 | 3.54E-16 | down |
| hsa-mir-130a | -1.003618453 | 6.007195226 | 2.92E-16 | 4.02E-15 | down |
| hsa-mir-33b | -1.496203711 | 3.358481384 | 3.88E-16 | 5.13E-15 | down |
| hsa-mir-195 | -1.143046473 | 5.29479537 | 1.63E-14 | 1.58E-13 | down |
| hsa-mir-326 | -1.17741738 | 2.406007205 | 6.33E-14 | 5.64E-13 | down |
| hsa-mir-6503 | -1.200305457 | -0.154786921 | 1.06E-12 | 8.13E-12 | down |
| hsa-let-7c | -1.023305376 | 11.39523982 | 1.53E-11 | 1.03E-10 | down |
| hsa-mir-621 | -1.699895329 | -0.424992564 | 4.15E-11 | 2.59E-10 | down |
| hsa-mir-3614 | -1.044776688 | 2.27845851 | 7.86E-11 | 4.59E-10 | down |
| hsa-mir-199b | -1.228563505 | 10.14832722 | 1.34E-10 | 7.39E-10 | down |
| hsa-mir-6502 | -1.138479999 | -0.49054024 | 1.37E-09 | 6.32E-09 | down |
| hsa-mir-199a-2 | -1.137967739 | 10.04840894 | 4.19E-09 | 1.70E-08 | down |
| hsa-mir-199a-1 | -1.069772355 | 9.424282993 | 5.51E-08 | 1.87E-07 | down |
| hsa-mir-214 | -1.010921637 | 4.069093382 | 6.27E-07 | 1.82E-06 | down |
| hsa-mir-5589 | -1.188659428 | 4.397896875 | 1.47E-05 | 3.52E-05 | down |

**UP**: upregulated in HCC samples. **Down**: downregulated in HCC samples.
