# Supplementary material for: Identification of Prognostic Biomarkers and Correlation With Immune Infiltrates in Hepatocellular Carcinoma Based on a Competing Endogenous RNA Network
Source: Front Genet. 2021 May 20;12:591623. doi: 10.3389/fgene.2021.591623 (PMC8173128; doi:10.3389/fgene.2021.591623)
Supplement: Supplementary file 12 [file Table_2.DOCX]

**Table S2.** The 42 differentially expressed miRNAs significantly associated with survival time of HCC patients based on univariate COX regression analysis.

| **miRNA name** | **HR** | **P value** |
| --- | --- | --- |
| hsa-mir-139 | 0.71849283 | 5.86E-06 |
| hsa-mir-9-1 | 1.155223246 | 0.000322955 |
| hsa-mir-9-2 | 1.154796824 | 0.00032497 |
| hsa-mir-3677 | 1.355928322 | 0.000390053 |
| hsa-mir-9-3 | 1.152979895 | 0.000409679 |
| hsa-mir-105-1 | 1.100060804 | 0.000529817 |
| hsa-mir-3682 | 1.405576225 | 0.000577819 |
| hsa-mir-561 | 1.238968507 | 0.000902945 |
| hsa-mir-5003 | 1.412341908 | 0.001033867 |
| hsa-let-7c | 0.795881942 | 0.001109576 |
| hsa-mir-105-2 | 1.094989677 | 0.001124378 |
| hsa-mir-767 | 1.087666367 | 0.001555262 |
| hsa-mir-195 | 0.771789293 | 0.001738946 |
| hsa-mir-137 | 1.178763194 | 0.002137865 |
| hsa-mir-548f-1 | 1.321207667 | 0.002315361 |
| hsa-mir-3680-1 | 1.396062921 | 0.002897592 |
| hsa-mir-3189 | 1.247326995 | 0.003499997 |
| hsa-mir-3660 | 1.265161017 | 0.003616731 |
| hsa-mir-301a | 1.322354003 | 0.004291441 |
| hsa-mir-1180 | 1.259163522 | 0.004496278 |
| hsa-mir-4661 | 1.2174013 | 0.005163862 |
| hsa-mir-31 | 1.16164434 | 0.008407551 |
| hsa-mir-3074 | 1.269151848 | 0.009017517 |
| hsa-mir-326 | 1.211287006 | 0.012612547 |
| hsa-mir-3607 | 0.83119031 | 0.013001214 |
| hsa-mir-188 | 1.28085196 | 0.014534151 |
| hsa-mir-5010 | 1.29658544 | 0.014716296 |
| hsa-mir-877 | 1.23103479 | 0.015475839 |
| hsa-mir-3200 | 1.149780218 | 0.018094718 |
| hsa-mir-621 | 0.764217814 | 0.018573204 |
| hsa-mir-30d | 0.790821623 | 0.018755003 |
| hsa-mir-6844 | 1.239227615 | 0.026216266 |
| hsa-mir-4746 | 1.204239258 | 0.029646549 |
| hsa-mir-421 | 1.248578972 | 0.029836016 |
| hsa-mir-4652 | 1.082319321 | 0.030477112 |
| hsa-mir-3127 | 1.270112683 | 0.033481962 |
| hsa-mir-3923 | 1.071553236 | 0.040992263 |
| hsa-mir-3922 | 1.208889043 | 0.041433706 |
| hsa-mir-34a | 0.816328712 | 0.04320222 |
| hsa-mir-18a | 1.167751992 | 0.043315472 |
| hsa-mir-147b | 1.172288981 | 0.044161851 |
| hsa-mir-3614 | 0.851766796 | 0.049625111 |
