# Supplementary material for: Identification of Prognostic Biomarkers and Correlation With Immune Infiltrates in Hepatocellular Carcinoma Based on a Competing Endogenous RNA Network
Source: Front Genet. 2021 May 20;12:591623. doi: 10.3389/fgene.2021.591623 (PMC8173128; doi:10.3389/fgene.2021.591623)
Supplement: Supplementary file 13 [file Table_3.DOCX]

**Table S3**. The 23 differentially expressed miRNAs after LASSO analysis in HCC cohort.

| **DEmiRNA** | **logFC** | **logCPM** | **PValue** | **FDR** | **Differential type** |
| --- | --- | --- | --- | --- | --- |
| hsa-mir-105-1 | 8.635895124 | 4.32926991 | 1.69E-16 | 2.56E-15 | UP |
| hsa-mir-9-1 | 3.600281942 | 8.666350826 | 2.51E-16 | 3.69E-15 | UP |
| hsa-mir-30d | 1.288524235 | 13.93333531 | 2.68E-16 | 3.85E-15 | UP |
| hsa-mir-9-2 | 3.567063331 | 8.669445504 | 4.42E-16 | 5.72E-15 | UP |
| hsa-mir-4661 | 1.997228538 | 2.945685159 | 2.00E-15 | 2.27E-14 | UP |
| hsa-mir-301a | 1.315970189 | 3.289789811 | 6.45E-15 | 6.95E-14 | UP |
| hsa-mir-3923 | 7.523074791 | 3.947550032 | 2.81E-12 | 2.04E-11 | UP |
| hsa-mir-5010 | 1.133352197 | 0.560199532 | 2.47E-10 | 1.30E-09 | UP |
| hsa-mir-5003 | 1.611643075 | -0.29454556 | 1.18E-09 | 5.56E-09 | UP |
| hsa-mir-3682 | 1.154337561 | 1.022300534 | 1.25E-09 | 5.85E-09 | UP |
| hsa-mir-137 | 4.06796214 | 0.554895043 | 4.11E-08 | 1.41E-07 | UP |
| hsa-mir-6844 | 2.151366371 | -0.562662907 | 9.61E-08 | 3.11E-07 | UP |
| hsa-mir-548f-1 | 4.606963969 | 0.248887853 | 4.15E-07 | 1.24E-06 | UP |
| hsa-mir-3680-1 | 1.408298587 | -0.644348237 | 3.84E-06 | 9.85E-06 | UP |
| hsa-mir-561 | 2.565961815 | -0.266809771 | 1.20E-05 | 2.89E-05 | UP |
| hsa-mir-3660 | 2.862077303 | -0.731497014 | 0.00013017 | 0.000276246 | UP |
| hsa-mir-139 | -1.500161031 | 7.46632081 | 2.91E-26 | 1.92E-24 | down |
| hsa-mir-3607 | -1.656508447 | 6.435093514 | 4.97E-21 | 1.37E-19 | down |
| hsa-mir-195 | -1.143046473 | 5.29479537 | 1.63E-14 | 1.58E-13 | down |
| hsa-mir-326 | -1.17741738 | 2.406007205 | 6.33E-14 | 5.64E-13 | down |
| hsa-let-7c | -1.023305376 | 11.39523982 | 1.53E-11 | 1.03E-10 | down |
| hsa-mir-621 | -1.699895329 | -0.424992564 | 4.15E-11 | 2.59E-10 | down |
| hsa-mir-3614 | -1.044776688 | 2.27845851 | 7.86E-11 | 4.59E-10 | down |

UP: upregulated in HCC samples. Down: downregulated in HCC samples.
