# Supplementary material for: Identification of Prognostic Biomarkers and Correlation With Immune Infiltrates in Hepatocellular Carcinoma Based on a Competing Endogenous RNA Network
Source: Front Genet. 2021 May 20;12:591623. doi: 10.3389/fgene.2021.591623 (PMC8173128; doi:10.3389/fgene.2021.591623)
Supplement: Supplementary file 14 [file Table_4.DOCX]

Table S4. The differently expressed miRNAs (DEMs) related to overall survival for HCC patients with statistical significance of P value < 0.05.

| miRNA | logFC | logCPM | PValue | FDR | DE |
| --- | --- | --- | --- | --- | --- |
| hsa-mir-9-1 | 3.600281942 | 8.666350826 | 2.51E-16 | 3.69E-15 | UP |
| hsa-mir-9-2 | 3.567063331 | 8.669445504 | 4.42E-16 | 5.72E-15 | UP |
| hsa-mir-561 | 2.565961815 | -0.266809771 | 1.20E-05 | 2.89E-05 | UP |
| hsa-mir-6844 | 2.151366371 | -0.562662907 | 9.61E-08 | 3.11E-07 | UP |
| hsa-mir-301a | 1.315970189 | 3.289789811 | 6.45E-15 | 6.95E-14 | UP |
| hsa-mir-30d | 1.288524235 | 13.93333531 | 2.68E-16 | 3.85E-15 | UP |
| hsa-mir-3682 | 1.154337561 | 1.022300534 | 1.25E-09 | 5.85E-09 | UP |
| hsa-mir-5010 | 1.133352197 | 0.560199532 | 2.47E-10 | 1.30E-09 | UP |
| hsa-mir-195 | -1.143046473 | 5.29479537 | 1.63E-14 | 1.58E-13 | down |
| hsa-mir-326 | -1.17741738 | 2.406007205 | 6.33E-14 | 5.64E-13 | down |
| hsa-mir-139 | -1.500161031 | 7.46632081 | 2.91E-26 | 1.92E-24 | down |

UP: upregulated in HCC samples. Down: downregulated.
