# Supplementary material for: Identification of Prognostic Biomarkers and Correlation With Immune Infiltrates in Hepatocellular Carcinoma Based on a Competing Endogenous RNA Network
Source: Front Genet. 2021 May 20;12:591623. doi: 10.3389/fgene.2021.591623 (PMC8173128; doi:10.3389/fgene.2021.591623)
Supplement: Supplementary file 15 [file Table_5.DOCX]

**Table S5.** The differently expressed genes (DEGs) in HCC cohort associated with ceRNA network.

| **Gene name** | **Gene symbol** | **logFC** | **logCPM** | **PValue** | **FDR** | **Difference** |
| --- | --- | --- | --- | --- | --- | --- |
| LIN28B | ENSG00000187772 | 9.4782063 | 1.1492929 | 2.11E-14 | 1.67E-13 | UP |
| FOXG1 | ENSG00000176165 | 5.6869061 | -2.212304 | 7.05E-07 | 2.00E-06 | UP |
| HOXA10 | ENSG00000253293 | 5.659293 | 1.2242712 | 1.79E-23 | 3.87E-22 | UP |
| SALL3 | ENSG00000256463 | 5.5726773 | -2.154865 | 8.58E-07 | 2.41E-06 | UP |
| MYBL2 | ENSG00000101057 | 4.6029065 | 4.2776052 | 1.33E-40 | 1.42E-38 | UP |
| CCNE1 | ENSG00000105173 | 4.0755189 | 2.7869251 | 4.81E-26 | 1.39E-24 | UP |
| MKRN3 | ENSG00000179455 | 4.025649 | -0.632566 | 5.99E-17 | 6.57E-16 | UP |
| DEPDC1 | ENSG00000024526 | 3.8108178 | 2.1449156 | 1.07E-38 | 9.37E-37 | UP |
| CELSR3 | ENSG00000008300 | 3.559344 | 2.6054186 | 1.54E-39 | 1.48E-37 | UP |
| E2F7 | ENSG00000165891 | 3.5337286 | 0.7741983 | 1.07E-31 | 5.03E-30 | UP |
| USH1G | ENSG00000182040 | 3.4712665 | -2.745194 | 5.63E-08 | 1.88E-07 | UP |
| SIX4 | ENSG00000100625 | 3.4123619 | 0.2483056 | 6.03E-23 | 1.24E-21 | UP |
| KIF23 | ENSG00000137807 | 3.3451539 | 2.4774514 | 9.34E-39 | 8.25E-37 | UP |
| CEP55 | ENSG00000138180 | 3.0906604 | 1.7930027 | 3.10E-32 | 1.56E-30 | UP |
| HOXA3 | ENSG00000105997 | 2.8989086 | 1.3722489 | 2.77E-16 | 2.79E-15 | UP |
| CLSPN | ENSG00000092853 | 2.8467357 | 0.725935 | 1.91E-24 | 4.52E-23 | UP |
| CBX2 | ENSG00000173894 | 2.7429917 | 2.2037502 | 1.02E-27 | 3.38E-26 | UP |
| RACGAP1 | ENSG00000161800 | 2.4768631 | 3.6965908 | 3.70E-47 | 6.87E-45 | UP |
| PRRX1 | ENSG00000116132 | 2.4429715 | 1.4945284 | 1.36E-16 | 1.43E-15 | UP |
| ACSL4 | ENSG00000068366 | 2.2233559 | 7.9841171 | 1.09E-11 | 6.10E-11 | UP |
| CDC25A | ENSG00000164045 | 2.1923034 | 1.8895781 | 8.64E-22 | 1.56E-20 | UP |
| AXIN2 | ENSG00000168646 | 2.1583051 | 3.394623 | 1.16E-08 | 4.23E-08 | UP |
| ITGA2 | ENSG00000164171 | 2.0204843 | 3.0129069 | 1.28E-14 | 1.04E-13 | UP |
| RET | ENSG00000165731 | -2.1825784 | 1.2691019 | 1.82E-14 | 1.45E-13 | down |
| CPEB3 | ENSG00000107864 | -2.2009962 | 3.8843312 | 4.07E-55 | 1.71E-52 | down |
| SOCS3 | ENSG00000184557 | -2.2872764 | 5.5372798 | 8.64E-37 | 6.64E-35 | down |
| ESR1 | ENSG00000091831 | -2.4100791 | 3.5975272 | 1.01E-25 | 2.79E-24 | down |
| FOS | ENSG00000170345 | -3.0675479 | 6.9038714 | 1.56E-59 | 9.22E-57 | down |

**UP**: upregulated. **Down**: downregulated.
