# Supplementary material for: Identification of Prognostic Biomarkers and Correlation With Immune Infiltrates in Hepatocellular Carcinoma Based on a Competing Endogenous RNA Network
Source: Front Genet. 2021 May 20;12:591623. doi: 10.3389/fgene.2021.591623 (PMC8173128; doi:10.3389/fgene.2021.591623)
Supplement: Supplementary file 16 [file Table_6.DOCX]

**Table S6**. The interaction between genes in lncRNA-miRNA-mRNA network in HCC cohort.

| **mRNA** | **miRNA** | **lncRNA** |
| --- | --- | --- |
| AXIN2, HOXA3,KIF23,CPEB3 ITGA2, CCNE1, HOXA10, CEP55,CLSPN, CDC25A, CBX2, RET, E2F7 | hsa-mir-195 | PART1, C2orf48, CCDC13-AS1, AL033381.1, AP002478.1, FAM87A, WT1-AS, TCL6, AC087392.1, AC006305.1, AC016773.1, LINC00473, WARS2-IT1, SFTA1P, LINC00355, LINC00200, LINC00160, DLX6-AS1, BPESC1, DSCR10, TSPEAR-AS1, GPC6-AS1, CLRN1-AS1, MYLK-AS1, HOTTIP, GDNF-AS1, PVT1, RMST,LINC00485 |
| PRRX1 | hsa-mir-9-1 | PART1, CCDC13-AS1, AL357153.1, AL033381.1, WT1-AS, LINC00221, TCL6, AL512652.1, LINC00114, SFTA1P, LINC00392, HOTAIR, CCDC26, LINC00200, ERVH48-1, LINC00462, MYLK-AS1, CRNDE, PVT1,GRM5-AS1, RMST, AC040173.1, LINC00494, TSPEAR-AS1.LINC00501, AL163952.1 |
| MYBL2, LIN28B, SOCS3 MKRN3, CBX2, CELSR3 FOXG1, SIX4 | hsa-mir-30d | PART1, AP002478.1, FAM87A, WT1-AS, TCL6, AC087392.1, AC006305.1, LINC00114, HOTAIR, AC040173.1, ERVMER61-1, BPESC1, ERVH48-1, LINC00494, HOTTIP, PVT1,RMST, |
| PRRX1 | hsa-mir-9-2 | PART1, CCDC13-AS1, AL357153.1, AL033381.1, WT1-AS, LINC00221, TCL6, AL512652.1, LINC00114, SFTA1P, LINC00392, HOTAIR, CCDC26, LINC00200, ERVH48-1, LINC00462, MYLK-AS1, CRNDE, PVT1,GRM5-AS1, RMST, AC040173.1, LINC00494, TSPEAR-AS1, LINC00501, AL163952.1 |
| FOS | hsa-mir-139 | C2orf48, AL357153.1, WT1-AS, TCL6, AC087392.1, LINC00488, HAO2-IT1 MIR137HG, DLX6-AS1, BPESC1, PVT1, LINC00491, GRM5-AS1, RMST |
| RACGAP1, CEP55, ESR1 SALL3,DEPDC1,ACSL4, USH1G | hsa-mir-301a | PART1, CCDC13-AS1, AL357153.1, AL033381.1, FAM87A, C17orf82, LINC00221 TCL6, AL512652.1, LINC00501, LINC00272, AL359878.1, SOX21-AS1, HOTAIR ERVH48-1, AL139002.1, HOTTIP, AC068756.1,RMST |
