# Supplementary material for: Identification of Prognostic Biomarkers and Correlation With Immune Infiltrates in Hepatocellular Carcinoma Based on a Competing Endogenous RNA Network
Source: Front Genet. 2021 May 20;12:591623. doi: 10.3389/fgene.2021.591623 (PMC8173128; doi:10.3389/fgene.2021.591623)
Supplement: Supplementary file 17 [file Table_7.DOCX]

**Table S7.** Representative interactions of lncRNA-miRNA-hub genes in HCC.

| **mRNA** | **miRNA** | **lncRNA** |
| --- | --- | --- |
| CLSPN, CEP55 KIF23 | hsa-mir-195 | PART1, C2orf48, CCDC13-AS1, AL033381.1, AP002478.1, FAM87A, WT1-AS, TCL6, AC087392.1, AC006305.1, AC016773.1, LINC00473, WARS2-IT1, SFTA1P, LINC00355, LINC00200, LINC00160, DLX6-AS1, BPESC1, DSCR10, TSPEAR-AS1, GPC6-AS1,CLRN1-AS1, MYLK-AS1, HOTTIP, GDNF-AS1, PVT1, RMST, LINC00485 |
| CEP55, DEPDC1 RACGAP1 | hsa-mir-301a | PART1, CCDC13-AS1, AL357153.1, AL033381.1, FAM87A, C17orf82, LINC00221, TCL6, AL512652.1, LINC00501, LINC00272, AL359878.1, SOX21-AS1, HOTAIR, ERVH48-1, AL139002.1, HOTTIP, AC068756.1, RMST |
| MYBL2 | hsa-mir-30d | PART1, AP002478.1, FAM87A, WT1-AS, TCL6, AC087392.1, AC006305.1, LINC00114, HOTAIR, ERVMER61-1, BPESC1, ERVH48-1, LINC00494, HOTTIP, PVT1, RMST AC040173.1 |
