# Supplementary material for: Identification of Prognostic Biomarkers and Correlation With Immune Infiltrates in Hepatocellular Carcinoma Based on a Competing Endogenous RNA Network
Source: Front Genet. 2021 May 20;12:591623. doi: 10.3389/fgene.2021.591623 (PMC8173128; doi:10.3389/fgene.2021.591623)
Supplement: Supplementary file 18 [file Table_8.DOCX]

**Supplementary figure legends**

**Figure S1.** **The least absolute shrinkage and selection operator (LASSO) model was established based on the optimal penalty parameter.**

**Figure S2. The overall survival analysis of 11 DEmiRNAs with statistically significant, P value<0.05.** (A) hsa-mir-9-1. (B) hsa-mir-5010. (C) hsa-mir-6844. (D) hsa-mir-9-1. (E) hsa-mir-30d. (F) hsa-mir-139. (G) hsa-mir-195. (H) hsa-mir-301a. (I) hsa-mir-326. (J) hsa-mir-561. (K) hsa-mir-3682. The high expressed miRNAs were significantly correlated with shorter overall survival time in patients

**Figure S3. Heatmap of the significant signaling pathways enriched by the 11 DEmiRNAs based on DIANA-miRPath database.** The label in the right side represents the mature miRNA with corresponding to hsa-mir-9-2, has-mir-9-1, hsa-mir-6844, hsa-mir-3682, hsa-mir-139, hsa-mir-5010, hsa-mir-30d, hsa-mir-195, hsa-mir-301a, hsa-mir-326, hsa-mir-561.

**Figure S4. Ven diagram based on the intersection of DEGs in HCC cohort and predicted target genes of DEmiRNAs.**

**Figure S5. The functional enrichment analysis of GO and pathway for hub genes. A**: biological process (BP). **B**: cellular component (CC). **C**: molecular function (MF). **D**: KEGG pathway. **E**: Reactome pathway.

**Figure S6. The correlation between six hub genes of CEP55, CLSPN, DEPDC1, KIF23, MYBL2 and RACGAP1, and clinical prognosis in HCC**. (A-F) represents overall survival (OS) and (G-L) indicates recurrence-free survival (RFS) from TCGA cohort in GEPIA. (M-R) represents OS from five comprehensive databases (HGNC, NCBI, Ensembl, Uniprot, GeneCards) intergrated in the TISIDB.

**Figure S7. The transcriptional expression of hub genes in various tumor types by TIMER database** (*P < 0.05, **P <0.01, ***P < 0.001). **A**: CEP55. **B**: KIF23. **C**: CLSPN. D:MYBL2. **E**: DEPDC1. **F**: RAGCAP1.

**Figure S8. The correlation between the expression of hub genes and immune infiltration in HCC based on TIMER database.** (A) CEP55. (B) CLSPN. (C)DEPDC1. (E) KIF23. (F) MYBL2. (G) RACGAP1.

**Figure S9. The infiltrating level of each immune subset at different copy number status of hub genes in HCC.**

**Figure S10. The 3D structures of predicted bioactive compounds targeted hub genes via Drugbank database.** A: Irdabisant. B: CEP-9722. C: CEP-1347. D: CEP-37440. E: Cefapirin. F: Calusterone. G: Clotrimazole. H: Propafenone. I: Letrozole. J: Sildenafil. K: Ranitidine. L: Valproic acid. M: Esomeprazole. N: Pregabalin. The 3D structure of Calfactant didn’t be detected in database.
